# Ecology of Middle East respiratory syndrome coronavirus, 2012–2020: A machine learning modelling analysis

**DOI:** 10.1111/tbed.14548

**Published:** 2022-04-12

**Authors:** An‐Ran Zhang, Xin‐Lou Li, Tao Wang, Kun Liu, Ming‐Jin Liu, Wen‐Hui Zhang, Guo‐Ping Zhao, Jin‐Jin Chen, Xiao‐Ai Zhang, Dong Miao, Wei Ma, Li‐Qun Fang, Yang Yang, Wei Liu

**Affiliations:** ^1^ Department of Epidemiology, School of Public Health, Cheeloo College of Medicine Shandong University Jinan China; ^2^ State Key Laboratory of Pathogen and Biosecurity Beijing Institute of Microbiology and Epidemiology Beijing China; ^3^ Department of Biostatistics, College of Public Health and Health Professions University of Florida Gainesville Florida USA; ^4^ Emerging Pathogens Institute University of Florida Gainesville Florida USA; ^5^ Department of Medical Research, Key Laboratory of Environmental Sense Organ Stress and Health of the Ministry of Environmental Protection PLA Strategic Support Force Medical Center Beijing China; ^6^ Department of Epidemiology, Ministry of Education Key Lab of Hazard Assessment and Control in Special Operational Environment, School of Public Health Air Force Medical University Xi'an China; ^7^ Department of Epidemiology Logistics College of Chinese People's Armed Police Forces Tianjin China

**Keywords:** machine learning, MERS‐CoV, Middle East respiratory syndrome, predicted map, risk factors

## Abstract

The ongoing enzootic circulation of the Middle East respiratory syndrome coronavirus (MERS‐CoV) in the Middle East and North Africa is increasingly raising the concern about the possibility of its recombination with other human‐adapted coronaviruses, particularly the pandemic SARS‐CoV‐2. We aim to provide an updated picture about ecological niches of MERS‐CoV and associated socio‐environmental drivers. Based on 356 confirmed MERS cases with animal contact reported to the WHO and 63 records of animal infections collected from the literature as of 30 May 2020, we assessed ecological niches of MERS‐CoV using an ensemble model integrating three machine learning algorithms. With a high predictive accuracy (area under receiver operating characteristic curve = 91.66% in test data), the ensemble model estimated that ecologically suitable areas span over the Middle East, South Asia and the whole North Africa, much wider than the range of reported locally infected MERS cases and test‐positive animal samples. Ecological suitability for MERS‐CoV was significantly associated with high levels of bareland coverage (relative contribution = 30.06%), population density (7.28%), average temperature (6.48%) and camel density (6.20%). Future surveillance and intervention programs should target the high‐risk populations and regions informed by updated quantitative analyses.

## 1 INTRODUCTION

Middle East respiratory syndrome (MERS), a respiratory infectious disease caused by the Middle East respiratory syndrome coronavirus (MERS‐CoV), was first discovered in Saudi Arabia in September 2012 (Zaki et al., 2012). Individuals infected with MERS‐CoV may experience none, mild or severe respiratory illnesses or death, with a case fatality ratio of about 34% (WHO, 2020). As of 30 May 2020, a total of 27 countries in the Middle East, North Africa, Europe, Northeast Asia and North America have reported 2562 laboratory‐confirmed MERS cases and 881 associated deaths, according to the World Health Organization (WHO) (WHO, 2020). The vast majority of MERS cases were reported by the Kingdom of Saudi Arabia (KSA), followed by the Republic of Korea where cases were almost exclusively reported from a big outbreak in 2015 induced by a single imported case (WHO, 2020). Frequent travellers and pilgrims from and to the Middle East have raised concerns about a global pandemic, given the lack of effective treatment and vaccine (Memish et al., 2014). In February 2018, WHO formally incorporated MERS‐CoV into the Research and Development Blueprint to promote research in this area (Mehand et al., 2018). The ongoing pandemic of the severe acute respiratory syndrome coronavirus 2 (SARS‐CoV‐2) further exacerbated this concern due to the possibility of recombination.

Current epidemiological studies suggest that human‐to‐human transmission of MERS‐CoV is inefficient, and the primary infection source is zoonotic (Anthony et al., 2017; David et al., 2018). While the number and size of outbreaks have decreased in recent years, the proportion of primary MERS‐CoV cases is gradually increasing (Zhang et al., 2021). A major driver for zoonotic infections is exposure to dromedaries or consumption of their raw products such as milk (Anthony et al., 2017; Chan et al., 2014; David et al., 2018), although other mammals may also serve as the reservoir (Alraddadi et al., 2016; David et al., 2018; Hui et al., 2018; Killerby et al., 2020). A recent study showed a significant correlation between primary MERS‐CoV cases and camel clusters at the provincial level in the KSA (Al‐Ahmadi et al., 2020). Besides, MERS‐CoV‐specific antibodies and MERS‐CoV RNA have been widely detected in camels across the Middle East and North, West and East Africa (Dighe et al., 2019; FAO‐OIE‐WHO MERS Technical Working Group, 2018; Sikkema et al., 2019). However, few studies have assessed the ecological suitability of the virus worldwide at a refined spatial resolution. The presence of an animal reservoir is a necessary but not sufficient condition for zoonotic diseases, as many other factors are needed for sustained spillover or endemic transmission. Similarly, reporting of opportunistic human cases does not imply establishment of an ecological niche as these sporadic cases could be imported. A rigorous assessment of the ecology of MERS‐CoV at the global scale is urgently needed to improve surveillance in high‐risk areas in preparation for a potential pandemic.

Machine learning (ML) techniques are increasingly popular in ecological studies (Bhatt et al., 2013; Fang et al., 2013; Sinka et al., 2010). By collecting up‐to‐date surveillance and contact tracing data from multi‐sources including the WHO, public health agencies and peer‐reviewed literature, we used several popular ML algorithms and their ensemble to assess the contributions of a variety of environmental, socioeconomic and biological factors to the ecological suitability of MERS‐CoV. We mapped the risk distribution predicted by the best model to indicate where this pathogen may invade and establish and where public health surveillance and interventions are most needed to prevent a potential pandemic.

## 2 METHOD

### 2.1 Data collection

**FIGURE 1:**
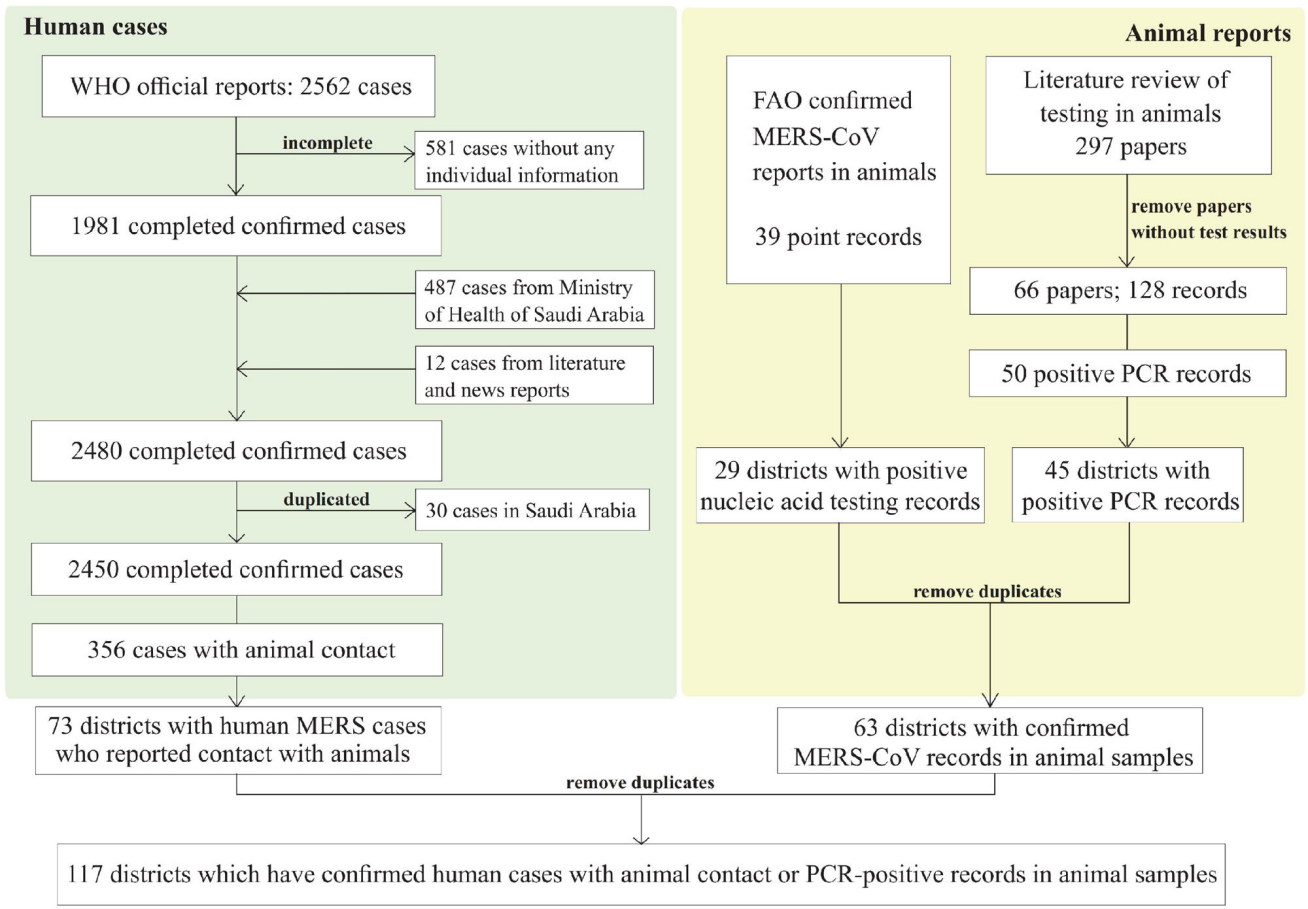
The flow chart for the selection of case districts that have evidence for presence of MERS‐CoV, that is human MERS cases with animal contact and PCR‐positive animal samples

**FIGURE 2:**
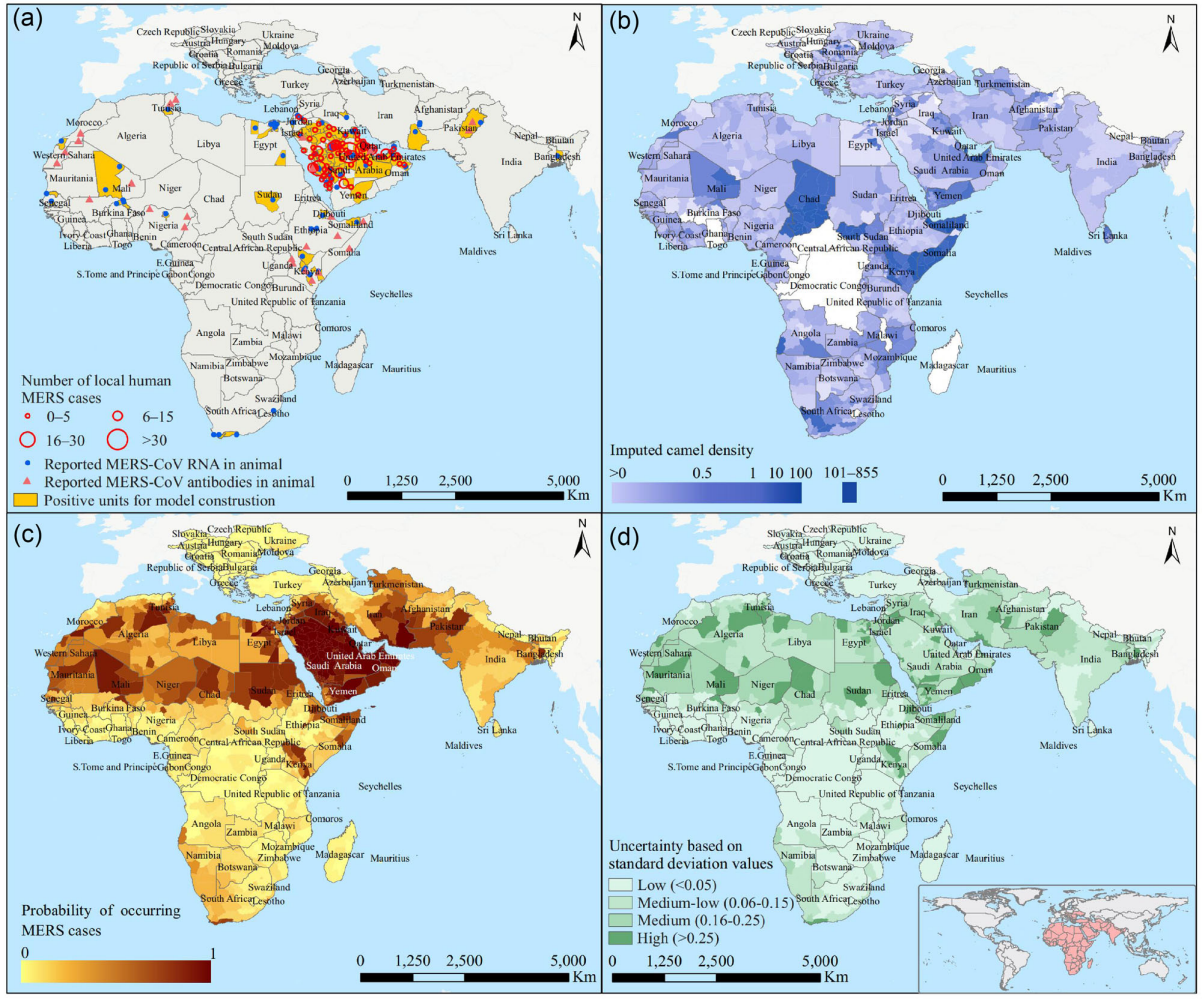
Ecological assessment for MERS‐CoV using the ensemble model. The spatial distribution of case districts with either human MERS cases with animal contact or MERS‐CoV‐positive animal samples is shown in panel a. The spatial distribution of camel densities by averaging over 100 imputed data sets is shown in panel b. Ensemble‐model‐predicted risks of MERS‐CoV presence were mapped in panel c, and the uncertainty levels (standard deviation of predicted risk values over the 100 imputed data sets) were mapped in panel d

Data on confirmed MERS cases were collected from official reports of WHO, the Food and Agriculture Organization (FAO) of the United Nations and the health departments of MERS‐affected countries, which were cross‐validated with and supplemented by data from a literature review. Search medical subject headings (MeSH) terms used were ‘Middle East Respiratory Syndrome Coronavirus/MERS‐CoV/HCoV‐EMC’ or ‘Middle East respiratory syndrome/Middle Eastern Respiratory Syndrome/MERS’. All the cases had been confirmed following a standard WHO technical guidance (https://www.who.int/csr/disease/coronavirus_infections/case_definition/en/) (Tables S1 and S6). Each confirmed case was geo‐referenced and mapped according to the finest address available using GIS technologies. Records of MERS‐CoV detection in animals, including detection dates, geographic locations and positive rates, were obtained from FAO and literature review. In addition, we conducted a PubMed search for papers published in English since 1 January 2012, using search terms (‘Middle East Respiratory Syndrome Coronavirus/ MERS‐CoV/HCoV‐EMC’ or ‘Middle East respiratory syndrome/Middle Eastern Respiratory Syndrome/MERS’) and (‘camel’ or ‘dromedary’ or ‘animal’) and (‘prevalence’ or ‘sample’ or ‘surveillance’ or ‘test’). Animal studies solely based on serological surveys were excluded because (1) specificity of existing serological tests is not clear, and (2) seropositivity does not necessarily reflect recent infection and the origin of infection is often obscured by frequent animal trade. The digital boundary maps of country‐level and district‐level data were obtained from the GADM database of the Global Administrative Areas version 2.0 (http://www.gadm.org) (Tables S1 and S7). We identified 73 districts with human cases who reported contact with animals or animal products and 63 districts with PCR‐positive animal samples (Figure 1), where district refers to the second‐level administrative spatial unit in most countries except for the KSA. We treat counties in the KSA as districts, because the second‐level administrative areas are provinces which are much larger than districts in other countries. After removing duplicates, in total 117 districts are considered as ‘case’ districts in our modelling practice (Figure 1). We then expanded the study region from countries with ‘case’ districts to incorporate the whole Africa continent, the Middle East, part of the West Asia and part of the Eastern Europe (shaded in Figure 2a) for ecological modelling. This choice of study region is to balance between diversity of ecological conditions and the possibility of MERS‐CoV spread via migration of animal hosts. Countries with opportunistic long‐distance importation by travellers such as the United Kingdom and South Korea are not included. A total of 1937 case districts were identified in our study regions (Figure 2a). The other 1820 districts in the study region without reported MERS human cases or MERS‐CoV‐positive animal specimens were considered as the pool of control districts (as of the end of May 2020). Socio‐environmental features that may potentially contribute to the ecology of MERS‐CoV were collected at the district level, including population density, camel density, monthly meteorological data, elevation, land cover, economic development level, transportation and locations of hospitals. All the data sources involved in this study are given in Table S1.

### 2.2 Feature imputation and selection

We obtained 34 variables (33 district‐level data and camel density data at district level after imputing) from all data sources (Table S2). Population densities of the raster type with a resolution of 30 arc seconds were downloaded from http://sedac.ciesin.columbia.edu/gpw/credits.jsp and were translated to average numbers of persons per square kilometre at the district level. Meteorological data including 19 ecoclimatic variables (Bio01‒19) were obtained from http://www.ncdc.noaa.gov/. Elevation data were collected from http://srtm.csi.cgiar.org and average altitude of each district was calculated. Raster type land cover data with a resolution of 300 m were collected from http://ionial.esrin.esa.int. Percentage coverages of cropland, forest, grassland, shrubland, wetland, built‐up land, bare land, water body and ice land were extracted from the land cover data and the average coverages at the district level were calculated. Major transportation routes including railways and main roads were collected from the OpenStreetMap project (http://download.geofabrik.de/). The intersections of major transportation routes with each district were identified. Data on locations of hospitals were collected from the OpenStreetMap project (http://download.geofabrik.de/) and zonal statistics were used to summarize the total numbers of hospitals at the district level. All spatial analyses and zonal statistics were performed in ArcGIS 10.5 (Esri Inc, Redlands, CA, USA).

We collected camel density data from three sources: (1) camel density data at the district level from 2012 to 2020 were obtained from the World Organization for Animal Health (OIE; https://wahis.oie.int/#/dashboards/qd‐dashboard); (2) camel count data at the district level in the KSA were obtained from a national agricultural census conducted by the General Authority for Statistics of the KSA (https://www.stats.gov.sa/en/22); and (3) for some countries without district‐level data, the total numbers of camels at the country level were obtained from the FAO (http://www.fao.org/faostat/en). The distribution of camel data availability is shown in Figure S3a. As camel is a major reservoir of the MERS‐CoV, we imputed missing camel densities at the district level (region either without camel density data or with only the total number of camels at the country level) using a Classification and Regression Trees (CART) model (Supporting Information).

### 2.3 Model development

The selected features were assessed for their association with the presence/absence of MERS human cases or MERS‐CoV‐positive animal specimens using several ML algorithms that have been widely applied in ecological studies (Bhatt et al., 2013; Fang et al., 2013; Shearer et al., 2018). In this study, a ‘case–control’ design was constructed to assess ecological features for their impacts on the presence/absence of MERS human cases or MERS‐CoV‐positive animal specimens at the district level. Before the model fitting, multicollinearity among features was screened via the Spearman rank correlation coefficient. For each group of highly correlated variables (correlation coefficient ≥.7), only one variable was chosen as the proxy to be used for fitting models (Figure S4).

A two‐stage model was developed in this study. In the first stage, we fitted three mainstream ML models including random forest (RF), boosted regression trees (BRT) and support vector machine (SVM) to the data, using R packages *randomForest*, *gbm* and *e1071*. For each model, the following bootstrapping‐and‐fitting steps were replicated 100 times, each using one of the 100 imputed data sets of camel densities. At first, to improve the robustness of the model and to avoid over fitting, we bootstrapped 100 ‘case’ districts from the overall pool of 117 ‘case’ districts reporting MERS cases or MERS‐CoV‐positive animal specimens. For each selected ‘case’, we bootstrapped four ‘controls’ from the pool of 1820 ‘control’ districts reporting neither MERS case nor positive animal specimen, to reach a total of 400 (22%) controls. The number of cases, 100, was chosen to be close to the total number of available case districts while leaving some space for randomness. The 1:4 case–control ratio is based on our previous ecological studies for avian influenza virus A (H7N9) (Fang et al., 2013) and severe fever with thrombocytopenia syndrome (SFTS) (Liu et al., 2015). Features that are time varying, for example, population density and meteorological variables, were averaged over the study period. Next, the bootstrap data set was randomly partitioned into a 75% training data set and a 25% testing data set. The base models were then built using the training data set and validated using the testing data set. Finally, the fitted models were used to predict the probability of MERS‐CoV presence for all districts in our data.

In the second stage, we built an ensemble model by stacking the three base ML models. Stacking is one of the ensemble learning strategies that became popular because of their more robust prediction than individual learners (Breiman, 1996; Wolpert, 1992). The model‐building procedure involves two steps. In the first step, all base ML models were trained on the training data set with three fold cross‐validation, from which we obtained a meta training data set with predicted probabilities as columns, one per each base model. The trained base models were also applied to the test data set to generate a meta‐test data set, also with predicted probabilities as columns, one per each base model. In the second step, we fitted an XGBoost model, called the meta‐learner, to the meta‐training data set, using the predicted probabilities from the base models as features. XGBoost is a scalable gradient tree boosting algorithm which improves the efficiency of split finding with weighted quantile sketch and is able to handle sparsity in features (Chen & Guestrin, 2016). It has been used for disease diagnosis and prediction (Davagdorj et al., 2020; Guan, et al., 2021; Ogunleye & Wang, 2020). The predictive performance of the meta‐learner was evaluated using the meta test data. Considering that our study is essentially based on presence‐only data as absence of MERS‐CoV in a given area is much harder to prove, a sensitivity analysis was conducted using the maximum entropy (MaxEnt) model, a popular tool for modelling presence‐only data (Li et al., 2011).

### 2.4 Model evaluation and risk mapping

Predictive performance of the models was assessed by a variety of indicators including areas under the receiver operating characteristic curve (AUC), accuracy, sensitivity, specificity, positive predictive value (PPV), negative predictive value (NPV), F1 and Kappa. Relative importance of each feature was averaged over the 100 bootstrap data sets for each method, and we report those given by the most predictive model as the primary results. The risk map of MERS was created based on the final average predicted probabilities from the stacking model using ArcGIS 10.5 (Esri Inc, Redlands, CA, USA).

### 2.5 Other statistical analyses

Comparisons between groups were conducted using Wilcoxon rank‐sum test for continuous variables and Fisher's exact test for categorical variables. Using predictive features found by the best ML model, we performed logistic regression on the presence of MERS‐CoV to evaluate their effects in terms of odds ratios (OR). All analysis was performed in R 3.6.2. All statistical tests were two‐sided at the significance level of .05.

## 3 RESULTS

From September 2012 to May 2020, a total of 2562 laboratory‐confirmed MERS human cases were reported. After excluding 112 cases with incomplete data and 2094 cases who did not report animal contact, 356 confirmed MERS cases and the districts they belong to were included in our final analysis (Figure 1). MERS cases who reported local animal contact history were all infected in the Middle East, although they could have travelled to and were identified in Europe and Southeast Asia (Zhang et al., 2021; Figure S1). Before 2019, there was a clear increasing trend in the proportion of cases with animal contact (Table S3). Compared to those without animal contact, these cases were significantly older, had more underlying conditions, had longer delay in diagnosis and were associated with higher CFR (Table S3).

As camel density is an important risk driver based on current knowledge but not available in many places, we performed statistical imputation of this variable. CART provided the highest *R* ^2^ for fitting the regression of camel density on other completely observed variables (Figure S2) and was thus used to generate 100 complete data sets for further modelling. The imputed camel densities match the observed data satisfactorily (Figures 2b and S3).

The three base models (boosted regression tree [BRT], support vector machine [SVM] and random forest [RF]) and the ensemble model that stacks all three showed satisfactory performance for predicting MERS‐CoV presence (Table 1 and Figure S5). The ensemble model attained the highest prediction efficiency with a mean AUC of 91.66% (95% CI: 89.16%‒94.15%) on the meta‐test data, followed by BRT with a mean AUC of 90.86% (95% CI: 88.05%‒93.67%). We selected cut‐off‐predicted values of 0.54, 0.47, 0.48 and 0.62 associated with the highest F1 score to assign the predicted risk label and calculate necessary inputs for the ensemble model (Figure S6). In addition to the highest AUC, the ensemble model was also associated with the highest accuracy (93.95%, 95% CI: 92.79%‒94.93%) and F1 score (0.54). RF provided a similar F1 score.

**TABLE 1:** Performance in risk prediction of MERS‐CoV presence for four models in training and test data sets. Results are averaged over 100 data sets with imputed camel densities

Model	AUC (95% CI, %)	Accuracy (95% CI, %)	Sensitivity (95% CI, %)	Specificity (95% CI, %)	F1 score	Kappa
Ensemble of 100 train data sets						
Ensemble	100 (100‒100)	100 (99.8‒100)	100 (96.82‒100)	99.79 (99.79‒100)	1	1
BRT	98.81 (98.06‒99.55)	98.61 (97.98‒99.04)	87.18 (79.92‒92.07)	98.85 (98.85‒99.62)	0.8831	0.8757
SVM	99.7 (99.51‒99.89)	98.71 (98.1‒99.12)	99.15 (95.32‒99.85)	98.05 (98.05‒99.11)	0.9027	0.8959
RF	100 (100‒100)	100 (99.8‒100)	100 (96.82‒100)	99.79 (99.79‒100)	1	1
Ensemble of 100 test data sets						
Ensemble	91.66 (89.16‒94.15)	93.95 (92.79‒94.93)	58.97 (49.91‒67.46)	95.24 (95.24‒97.01)	0.5433	0.5111
BRT	90.86 (88.05‒93.67)	92.48 (91.22‒93.58)	66.67 (57.72‒74.56)	92.98 (92.98‒95.16)	0.5200	0.4814
SVM	88.45 (85.13‒91.77)	91.13 (89.77‒92.32)	64.1 (55.09‒72.22)	91.6 (91.6‒93.98)	0.4688	0.4241
RF	84.14 (79.98‒88.3)	93.74 (92.56‒94.74)	60.68 (51.63‒69.06)	94.87 (94.87‒96.71)	0.5420	0.5088

Abbreviations: AUC, area under the receiver operating characteristics curve; BRT, boosted regression trees; CI, confidence interval; NPV, negative predictive value; PPV, positive predictive value; RF, random forest; SVM, support vector machine.

We mapped the average predicted probabilities of MERS‐CoV presence given by the ensemble model (Figure 2c). High and moderate risk areas span over the Middle East, West Asia and the whole North Africa, much wider than the geographic range with human MERS cases recorded. Southern Europe, eastern Africa and southern Africa were predicted to have mild risks, consistent with the observation of only a few human cases or positive animal samples in these regions. The risk map obtained from the maximum entropy MaxEnt model, another popular method for presence‐only data, also showed a comparable distribution (Figure S7). We estimated that 124.1 (95% CI: 110.7−137.6) million people live in high‐risk areas, accounting for 2% of the total global population. Uncertainty in the spatial predictions seems to be high in many high‐risk districts in northern Africa (Figure 2d), possibly due to lack of human cases.

### 3.1 Variable importance

**FIGURE 3:**
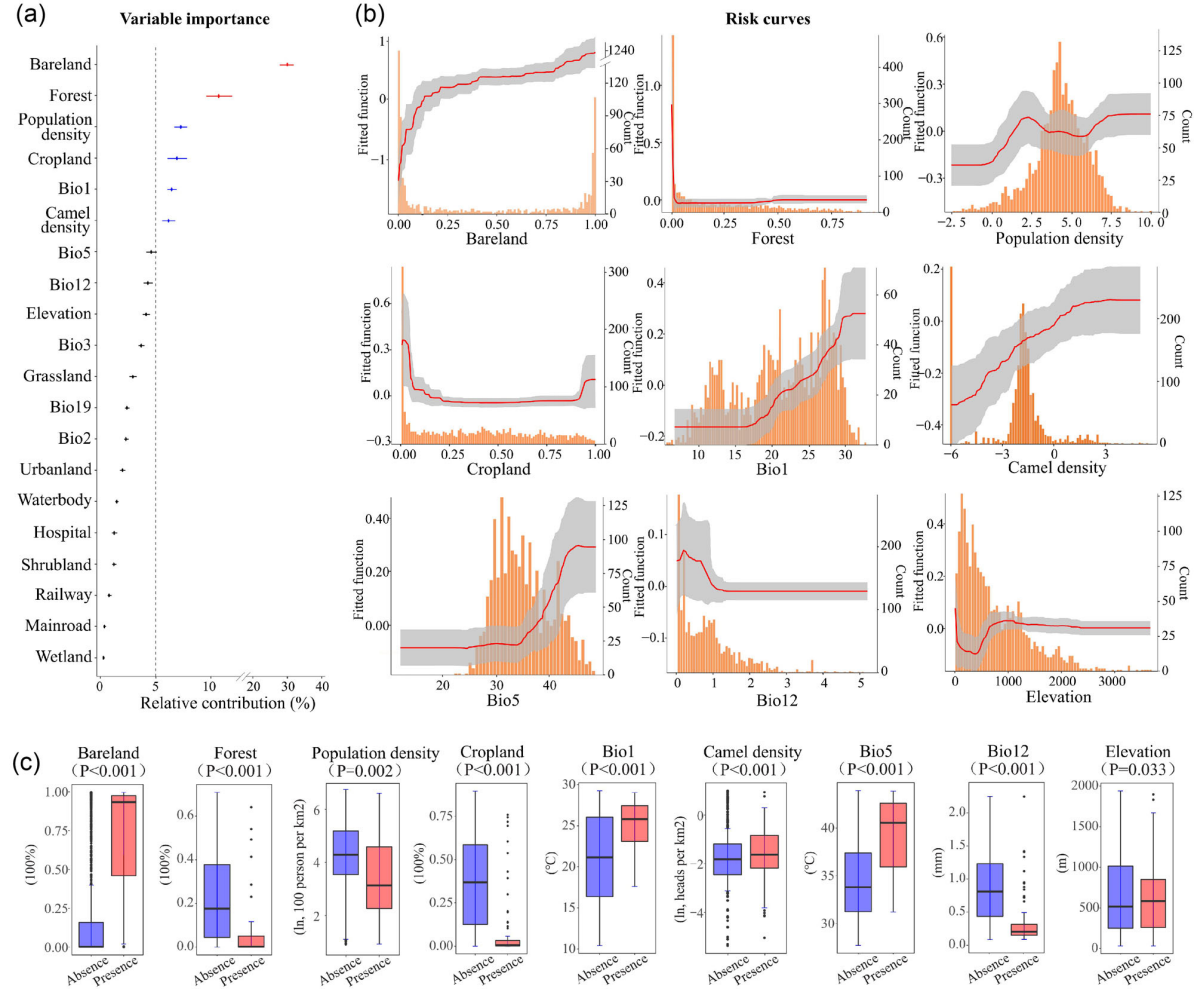
Contributions of key socioenvironmental factors to ecological suitability of MERS‐CoV identified by the BRT model. (a) The relative contributions of 20 variables included in the BRT model. Points represent the mean values, and bars represent the 95% confidence intervals. High (≥10%) and moderate (≥5%, <10%) relative contributions are coloured in red and blue, respectively. (b) Risk curves of the top nine influential factors. A higher risk value corresponds to a better ecological suitability for the virus. The distribution of each factor in the data is shown as the histograms in orange. (c) Box plots showing difference in the distribution of the top nine contributors between case districts (*n* = 117) and the pool of all control districts (*n* = 1820). Extreme values are removed. The centre line represents the median, and the box represent the inter‐quartile range. *P*‐values are based on Wilcoxon rank sum test except two‐sample *t*‐test is used for bareland coverage

Based on the BRT model, we found that bareland coverage was the leading contributor to the risk of MERS‐CoV presence, with a relative contribution (RC) of 30.06% (95% CI: 28.61%−31.50%), followed by coverage of forest land with an RC of 10.74% (95% CI: 9.56%−11.91%) (Figure 3a and Table S4). Population density, annual mean temperature (Bio1), coverage of cropland and camel density had moderate RCs, ranging from 6.2% to 7.28%. The higher risk of MERS‐CoV presence was associated with higher levels of bareland coverage, population density, annual mean temperature and camel density, but was associated with lower levels of forest coverage and cropland coverage (Figure 3b). The model‐based findings regarding the effects of the risk factors are mostly consistent with the univariable analyses for dichotomized factors except for population density (Figure 3c and Table S5). Among the top nine predictors selected by the BRT model, eight were also classified among the top nine contributors by the RF model. Besides, bareland coverage was also ranked the most important by the RF, SVM and MaxEnt models (Table S4 and Figure S7).

### 3.2 Logistic analysis based on extracted important variables

To interpret the effects of influential risk predictors using more traditional odds ratios, we conducted a logistic regression with the seven predictors (bareland coverage, forest coverage, population density, Bio1, cropland coverage, Bio5 and camel density) that have relative contributions over 5% in the BRT model (Table 2). These predictors were dichotomized based on their values associated with the best Youden index (Figure S8), except that population density was categorized into three levels based on inflection points (6.05 and 181.3) of the BRT‐estimated risk curve (Figure 3b). Forest coverage and cropland coverage were excluded from the multivariable logistic analysis for their high correlations with bareland coverage (*P* > .6). Bareland coverage, Bio1 and camel density were significantly associated with presence of MERS‐CoV in both the univariable and multivariable analyses (Table 2). Bareland (>8.12% vs. ≤8.12%) was associated with an OR of 23.74 (95% CI: 12.93‒43.58), and Bio1 (>20.48℃ vs. ≤20.48℃) was associated with an OR of 4.05 (95% CI: 2.13‒7.69). Furthermore, no significant two‐way interaction was found among bareland coverage, population density, Bio1 and camel density.

**TABLE 2:** Logistic regression analysis of risk factors associated with evidence of presence of MERS‐CoV for all study areas

Variables	Outcome (No.)		Univariate			Multivariate		
	Presence	Absence	OR	95% CI	*P*	OR	95% CI	*P*
Percentage coverage of bareland (%)								
Low (≤8.12)	13	1416	1			1		
High (>8.12)	104	404	28.04	15.59‒50.44	<.001	23.74	12.93‒43.58	<.001
Percentage coverage of forest (%)^a^								
Low (≤0.80)	104	725	1			–		
High (>0.80)	13	1095	0.08	0.05‒0.15	<.001	–	–	–
Population density (/km^2^)								
Low (≤6.05)	18	160	1			1		
Medium (6.06‒181.30)	75	1181	0.56	0.33‒0.97	.038	2.39	1.34‒4.28	.003
High (>181.30)	24	479	0.45	0.24‒0.84	.013	1.9	0.93‒3.87	.079
Percentage coverage of cropland (%)								
Low (≤4.19)	92	338	1					
High (>4.19)	25	1482	0.06	0.04‒0.10	<.001			
Annual mean temperature (Bio1,℃)								
Low (≤20.48)	12	841	1			1		
High (>20.48)	105	979	7.52	4.11‒17.36	<.001	4.05	2.13‒7.69	<.001
Camel density (/km^2^)^b^								
Low (≤0.26)	65	1456	1			1		
High (>0.26)	52	364	3.2	2.18‒4.69	<.001	1.8	1.16‒2.80	.009

Abbreviations: CI, confidence interval; OR, odds ratio.

^a^ Percentage coverage of forest and cropland were not included in multivariate analysis for their high correlations with bareland coverage (*P* > .6).

^b^ Univariable analysis of camel density and multivariable analyses for all variables are averaged over 100 data sets with imputed camel densities.

## 4 DISCUSSION

Based on geographic locations of both MERS cases with animal contact and MERS‐CoV test‐positive animal samples up to May 2020, we provided a systematic analysis on suitable ecological niches for MERS‐CoV using ML approaches. The modelling results indicate a wide strip across North Africa, the Middle East and West Asia as high‐risk regions for the presence of MERS‐CoV. We found that bareland coverage and forest coverage are the two leading determinants for the presence of MERS‐CoV, and population density, annual mean temperature and camel density also played important roles.

By stacking three highly competent ML models, we predicted high risk of MERS‐CoV in areas harbouring 124.1 million residents, which is much wider than the regions that had recorded human cases of MERS. This gap between model‐predicted risks and the observed distribution of MERS cases implies the possibility of substantial under‐detection of human cases, possibly subclinical or asymptomatic, and animal hosts in regions outside the Middle East (Liljander et al., 2016). A recent study in Kenya had confirmed the existence of asymptomatic MERS cases in camel handlers (Munyua et al., 2021), which supports our result suggesting that MERS‐CoV might cause no or mild disease but undetected in Africa. Wide circulation of MERS‐CoV in dromedary camels in North, East and West Africa has been reported (Alshukairi et al., 2018; Hemida et al., 2017; Miguel et al., 2017). A recent observational study on a cohort with occupational exposure to dromedary camels in Nigeria supports the conjecture that MERS‐CoV infections in Africa have been severely underestimated (Mok et al., 2020). Even in the Middle East where MERS‐CoV is known to be enzootic, zoonotic infections could have also been under‐detected as cases who reported animal contact account for a relatively small proportion of all reported cases (Ye et al., 2020). The risk map could help us with prioritizing resource‐limited high‐risk areas for surveillance, prevention and control efforts.

We found that both bareland coverage and forest coverage are the leading predictors for the ecological niche of MERS‐CoV. The effect of bareland coverage is stable and strong in both the univariable and multivariable analyses. The importance of bareland in the transmission of MERS‐CoV might be related to abundance of camels (Mirkena et al., 2018; Sikkema et al., 2019). However, the leading role of bareland coverage is robust to the adjustment for both camel density and temperature, suggesting the possibility that bareland might be ecologically suitable for a variety of other animal hosts not considered in our models, for example certain types of bats and alpacas (Hui et al., 2018; Killerby et al., 2020).

While dromedary camels are known to be the major intermediate host for MERS‐CoV (Al‐Ahmadi et al., 2020; Killerby et al., 2020; Munyua et al., 2021), camel density only moderately contributed to the ecological models. A possible explanation could be that the overall camel density used for modelling does not capture the distribution of dromedary camels. In addition, the large amount of missing camel densities might have attenuated the actual effect, despite our efforts in statistical imputation.

Our study was subject to several limitations. First, the passive surveillance of MERS in most countries only captured patients who sought medical care, likely missing a substantial number of subclinical or asymptomatic infections. Second, only a few data were available in the literature for test‐positive samples from camels, and the sampling was likely not random. Both under‐detection and biased sampling could have introduced bias into our analyses. In addition, unlike traditional statistical regressions, the ML models are not able to use random effects to handle spatial heterogeneity and spatial correlation unexplained by the measured ecological features. Nevertheless, these models are designed to accommodate complex interactions among and nonlinear effects of ecological features which often account for most spatial heterogeneity, as indicated by the high AUC values. Finally, the ecological modelling considered only presence and absence of evidence for human cases or test‐positive animal samples, ignoring the frequency of cases or animal samples which might provide additional information about ecological suitability for persistence.

A cross‐continental fine‐scale risk assessment study of ecological niches of the MERS was carried out based on multi‐elements, which has not been reported in the current reported literatures. The risk map can be used to guide future surveillance of MERS‐CoV in both animal and human hosts, especially in resource‐limited countries. One lesson we learned from the pandemic of COVID‐19 is that more active surveillance on the animal–human interface is crucial for early warning of evolutionary signatures of the virus that are indicative of its transmission efficiency both from animal to human and from human to human, so that potential pandemic can be prevented or controlled at the source. The risk map can also inform selection of sites for clinical trials to evaluate effectiveness of intervention products or programs such as candidate vaccines against MERS‐CoV (Folegatti et al., 2020).

## AUTHOR CONTRIBUTIONS

LQF, WL, YY and WM conceived and designed the study. ARZ, XLL and TW performed the main data collection and analyses under supervision of LQF, YY, WL and WM. KL, MJL, WHZ, GPZ, JJC, XAZ and DM helped with the analyses. ARZ wrote the first draft. WL, LQF and YY helped with finalizing the manuscript. All authors contributed to and approved the final version of the manuscript.

## CONFLICT OF INTEREST

The authors declare no conflict of interest.

## ETHICS STATEMENT

This study was approved by the institutional review board of the Beijing Institute of Microbiology and Epidemiology (Beijing, China, 20J009). All data were collected from publicly available sources. Data were de‐identified, and informed consent was waived.

## Supporting information

Supporting Information

## Data Availability

All data relevant to the study are included in the article or uploaded as Supporting Information.
