# Supplementary material for: Ecology of Middle East respiratory syndrome coronavirus, 2012–2020: A machine learning modelling analysis
Source: Transbound Emerg Dis. 2022 Apr 12;69(5):e2122–31. doi: 10.1111/tbed.14548 (PMC9526759; doi:10.1111/tbed.14548)

**Supplementary Appendix:**

A.R. Zhang, et al. Ecology of Middle East Respiratory Syndrome Coronavirus, 2012–2020: a machine learning modeling analysis

**Data collection and management**

We assembled a comprehensive database of all confirmed MERS cases up to May 30^th^, 2020, mainly according to the official reports released by WHO (https://www.who.int/emergencies/mers-cov/en/). A confirmed case is defined as a person with laboratory confirmation of MERS-CoV infection, irrespective of clinical signs and symptoms. According to the WHO Interim Guidance for Investigation of Cases of Human Infection with MERS-CoV (WHO/MERS/SUR/15.2 Revision 1), all MERS cases should be reported with the following essential information: Basic information and demographics (name, address, age, sex, occupation, date of symptom onset, date of initial clinic visit, etc.), exposure information (animal exposure, human exposure, travel history), testing methods, and clinical course, to inform the most effective international preparedness and response. The actual MERS case data available from WHO’s website include the basic demographic information (gender, age, reporting country, city of residence, with occupation of health care worker or not, underlying chronic conditions), date of critical events (such as symptoms onset, first hospitalization, laboratory confirmation, et al), and basic exposure information (exposure history to animal or consumption of their raw products such as mild, exposure history to confirmed MERS cases). For cases with key information missing, we retrieved complementary data from other publicly accessible sources, including the health departments of major MERS-affected countries, e.g., the Ministry of Health of the Kingdom of Saudi Arabia (KSA) (https://www.moh.gov.sa/en/CoronaNew/PressReleases/Pages/default.aspx), news reports, and academic articles. Cases with essential information missing (age, sex, type of exposure) or not laboratory-confirmed were excluded. Duplicated recorded were also removed. Records of detections of MERS-CoV in animals were obtained from the Food and Agriculture Organization of the United Nations (FAO, <http://empres-i.fao.org/eipws3g/)> and supplemented by literature review. According to World Organization for Animal Health, a laboratory-confirmed infection of MERS-CoV in camels is defined as isolation of MERS-CoV or identification of viral nucleic acids using RT-PCR in a sample from a dromedary camel, regardless of presence of clinical signs. Identification by viral nucleic acids should include detection of at least two specific genomic targets in a sample; or identification of a single target combined with sequencing of a secondary target; or identification of a single target combined with a positive rapid test of MERS-CoV antigens.^1^ In our study, a valid record of animal infection with MERS-CoV must include locality and date of detection, animal species, and positive rate (number of positive samples/ number of tested samples). Animal studies solely based on serological survey were excluded because (1) specificity of existing serological tests is not clear, and (2) seropositivity does not necessarily reflect recent infection and the origin of infection is often obscured by frequent animal trade. The digital boundary maps of country-level and district-level data were obtained from GADM database of Global Administrative Areas version 2.0 (http://www.gadm.org). Based on the geographic range of we then expanded the study region from countries with reported case or PCR-positive animal samples districts to incorporate the whole Africa continent, the Middle East, part of the West Asia, and part of the Eastern Europe (shaded in figure 2A) for ecological modeling. This choice of study region is to balance between diversity of ecological conditions and the possibility of MERS-CoV spread via migration of animal hosts. Countries with opportunistic long-distance importation by travelers such as UK and South Korea are not included.

Multi-source data on eco-geographical and socioeconomic variables (EGV) which possibly contributed to diffusion and persistence of MERS were collected (Table S1).Raster type of population density with a resolution of 30 arc seconds were derived from the Socioeconomic Data and Applications Centre (SEDAC) of the International Geographic Science Information (http://sedac.ciesin.columbia.edu/gpw/credits.jsp) and recalculated into density of human population (person per km^2^) at district level. Meteorological data including temperature and relative humidity during the study period were obtained from National Oceanic and Atmospheric Administration (NOCC, http://www.ncdc.noaa.gov/) and 19 ecoclimatic variables (Bio01‒19) were calculated to reflect the average level of temperature and precipitation that differed marginally across seasons as previous study (Table S3). ^2^ Elevation data were derived from the shuttle radar topography mission (SRTM) 90m DEM Digital Elevation Database ([http://srtm.csi.cgiar.org](http://srtm.csi.cgiar.org/)) and average altitude of each district was calculated. Raster type land cover data with a resolution of 300m were collected (http://ionial.esrin.esa.int). Percentage coverage of cropland, forest, grassland, shrubland, wetland, built-up land, bare land, water body and ice land were extracted from land cover data and calculated at the district level. Transportation line including railways and main roads were collected from the OpenStreetMap project (http://download.geofabrik.de/).The intersection relationship between transportation line and each district were demonstrated by spatial analysis. Data on locations of hospitals were collected from the OpenStreetMap project (http://download.geofabrik.de/) and zonal statistics was performed to summarize the total number of hospitals at district level. All spatial analysis and zonal statistics were performed in ArcGIS 10.5 (Esri Inc, Redlands, CA, USA). All spatial analysis and zonal statistics were performed in ArcGIS 10.5 (Esri Inc, Redlands, CA, USA).

Finally, a total of 33 potential explanatory variables (the camel density data is a combination data after we imputed the missing camel density data, described in the followed description “Imputing the missing camel density data”) were management at the district level in this step (Table S2).

**Imputing the missing camel density data**

We collected camel density data from three sources: (1) camel density data at the district level from 2012 to 2020 were obtained from the World Organization for Animal Health (OIE, [https://wahis.oie.int/#/dashboards/qd-dashboard](https://wahis.oie.int/#/dashboards/qd-dashboard)); (2) Camel count data at the district level in the KSA were obtained from a national agricultural census conducted by the General Authority for Statistics of the KSA (<https://www.stats.gov.sa/en/22>); and (3) For some countries without district-level data, the total numbers of camels at the country level were obtained from the FAO (<http://www.fao.org/faostat/en>). The distribution of camel data availability is shown in Figure S3(A). As camel is a major reservoir of the MERS-CoV, we imputed missing camel densities at the district level, where the imputation model was chosen from the following candidate methods: Classification and regression trees (CART)^3^, Random forest^4^, Predictive mean matching^5^ and Linear regression^6^. We fitted each of the four methods to regions with completely observed district-level data in R version 4.0.

A total number of 36 variables (including Bio01-19; percentage coverages of cropland, forest, grassland, shrubland, wetland, built-up land, bare land, water body and ice land; density of human population; elevation; railway, main road, hospital, camel density at country level, the latitude and longitude of center point for each district were firstly screened for multicollinearity using Spearman rank correlation. For each group of highly correlated variables (correlation coefficient ≥0.7), only one variable was retained for the imputation model. Briefly, percentage coverage of iceland and 13 Bio variables (Bio 3, 4, 6–11, 13, 15–18) were excluded due to their high correlations with other variables. The remaining 22 variables were used to identify the best imputation model out of four candidate methods: classification and regression trees, random forest, predictive mean matching, and linear regression. R-square (R^2^) was used for model selection. The R^2^ value for the four fitted imputation methods are 62.92 for CART, 60.90 for random forest, 62.36 for predictive mean matching, and 57.60 for linear regression (Figure S2). The CART yielded the highest R^2^ value and was chosen to impute the missing camel density data. We used the CART model to generate 100 complete data sets for subsequent ecological modeling for MERS-CoV. The average district-level camel densities of the 100 imputed data were displayed in Figure S3(B).

After the imputing camel data, we obtained 34 potential explanatory variables at the district level (Table S2) in our study region for predicting the risk for MERS.

**Machine-learning methods**

Machine learning techniques are increasingly used in developing models for predictions of animals and animal-borne diseases due to their flexibilities in handling nonlinearity, multi-way interactions, and different types of variables.^7 ,8-11^ In this study, three mainstream ML models, including random forest (RF), boosted regression trees (BRT) and support vector machine (SVM), were used to examine the association of the presence/absence of MERS-CoV (human cases ot test-positive animal specimens) with 34 selected features.

***Random forest (RF) model***

Random Forest is a robust machine learning algorithm that can be used for a variety of tasks including regression and classification^12^. It is itself an ensemble method, meaning that a random forest model is made up of a large number of small decision trees, called estimators, and each decision tree produces its own prediction. The random forest model combines the predictions of the estimators to produce a more accurate prediction^13^. When each tree is constructed, b samples are bootstrapped from the original training set of size N as the training subset and the remaining samples as the test subset. In this study, we set 500 trees and the remaining parameters are set to default values, by using R packages *randomForest*.

***Support vector machine (SVM) model***

An SVM model is basically a representation of different classes in a hyperplane in multidimensional space. The hyperplane will be generated in an iterative manner by SVM so that the error can be minimized^14^. The goal of SVM is to divide the datasets into classes to find a maximum marginal hyperplane (MMH). When given any set of labeled training data for separate categories, SVM can categorize new texts. It can perform to its optimum capacity when provided with limited data ^14^. For mainstream model, we trained the SVM system using a linear kernel. In our submissions, we set the penalty parameter C to 2 and the tolerance for stopping criteria to 1e-3 by using R packages *e1071,* and the remaining parameters are set to default values.

***BRT model***

BRT models are efficient for predicting distributions of organisms while accounting for non-linear covariate-response relationships and interactions between covariates, and therefore have been widely used to identify risk determinants for various zoonotic diseases^11,15^. It’s a stage-wise process to build a BRT model. At each stage, the optimal tree is found to explain the residuals of the model from the previous stage and is linearly combined with the existing trees. A bootstrap data set was drawn for tree-building at each stage to provide robust estimation of the model parameters. A tree complexity of 5, a learning rate of 0.005 and a bag fraction of 75% were used to identify the optimal tree for each bootstrap data. The relative weight for each variable was estimated from the identified trees and served as an indicator for the relative importance of that variable in predicting the outcome ^8,16^ by using R packages *gbm,* and the remaining parameters are set to default values.

***XGBoost model***

XGBoost is an implementation of Gradient Boosted decision trees^17^. In this algorithm, decision trees are created in a sequential fashion. Weighting plays an important role in XGBoost. Weights are assigned to all the independent variables which are then fed into the decision tree for predicting outcomes. These individual classifiers/predictors are then ensembled to give a more precise model. It can work on regression, classification, ranking, and user-defined prediction problems. XGBoosted model improves its efficiency in finding split points using weighted quantile sketch and is able to handle sparsity in features^17^. As a cutting-edge variation of gradient tree boosting, it has proven to push the limits of computing power for boosted trees algorithms, which has been used for disease diagnosis and prediction ^18-20^.

**Reference**

1. OIE. Middle East respiratory syndrome coronavirus (MERS-CoV) case definition for reporting to OIE. [https://www.oie.int/en/disease/middle-east-respiratory-syndrome-mers/.](http://www.oie.int/en/scientific-expertise/specific-information-and-recommendations/mers-cov/.) (accessed Feb 20, 2021).
2. Hijmans, R.J., Cameron, S.E., Parra, J.L., Jones, P.G. and Jarvis, A. (2005), Very high resolution interpolated climate surfaces for global land areas. *Int. J. Climatol.*, 25: 1965-1978. <https://doi.org/10.1002/joc.1276>
3. Hayes, T., Usami, S., Jacobucci, R., & McArdle, J. J. (2015). Using Classification and Regression Trees (CART) and random forests to analyze attrition: Results from two simulations. *Psychology and aging*, *30*(4), 911–929. <https://doi.org/10.1037/pag0000046>
4. Hong, S., & Lynn, H. S. (2020). Accuracy of random-forest-based imputation of missing data in the presence of non-normality, non-linearity, and interaction. *BMC medical research methodology*, *20*(1), 199. <https://doi.org/10.1186/s12874-020-01080-1>
5. Yang, S., & Kim, J. K. (2020). Asymptotic theory and inference of predictive mean matching imputation using a superpopulation model framework. *Scandinavian journal of statistics, theory and applications*, *47*(3), 839–861. <https://doi.org/10.1111/sjos.12429>
6. Beyad, Y., & Maeder, M. (2013). Multivariate linear regression with missing values. *Analytica chimica acta*, *796*, 38–41. <https://doi.org/10.1016/j.aca.2013.08.027>
7. Sinka, M. E., Rubio-Palis, Y., Manguin, S., Patil, A. P., Temperley, W. H., Gething, P. W., Van Boeckel, T., Kabaria, C. W., Harbach, R. E., & Hay, S. I. (2010). The dominant Anopheles vectors of human malaria in the Americas: occurrence data, distribution maps and bionomic précis. *Parasites & vectors*, *3*, 72. <https://doi.org/10.1186/1756-3305-3-72>
8. Elith, J., Leathwick, J. R., & Hastie, T. (2008). A working guide to boosted regression trees. *The Journal of animal ecology*, *77*(4), 802–813. <https://doi.org/10.1111/j.1365-2656.2008.01390.x>
9. Messina, J. P., Pigott, D. M., Golding, N., Duda, K. A., Brownstein, J. S., Weiss, D. J., Gibson, H., Robinson, T. P., Gilbert, M., William Wint, G. R., Nuttall, P. A., Gething, P. W., Myers, M. F., George, D. B., & Hay, S. I. (2015). The global distribution of Crimean-Congo hemorrhagic fever. *Transactions of the Royal Society of Tropical Medicine and Hygiene*, *109*(8), 503–513. <https://doi.org/10.1093/trstmh/trv050>
10. Randolph S. E. (2001). The shifting landscape of tick-borne zoonoses: tick-borne encephalitis and Lyme borreliosis in Europe. *Philosophical transactions of the Royal Society of London. Series B, Biological sciences*, *356*(1411), 1045–1056. <https://doi.org/10.1098/rstb.2001.0893>
11. Fang, L. Q., Li, X. L., Liu, K., Li, Y. J., Yao, H. W., Liang, S., Yang, Y., Feng, Z. J., Gray, G. C., & Cao, W. C. (2013). Mapping spread and risk of avian influenza A (H7N9) in China. *Scientific reports*, *3*, 2722. <https://doi.org/10.1038/srep02722>
12. Breiman, L., (2001).Random forests. *Machine learning.* 45(1): 5-32
13. Liaw, A., (2010). Documentation for R package randomForest. https://rdocumentation.org/packages/randomForest/versions/4.7-1.Accessed 1 June, 2020.
14. Cortes, C., Vapnik, V.,(1995) Support-vector networks. *Machine learning*. 20(3): 273-297
15. Gilbert, M., Golding, N., Zhou, H., Wint, G. R., Robinson, T. P., Tatem, A. J., Lai, S., Zhou, S., Jiang, H., Guo, D., Huang, Z., Messina, J. P., Xiao, X., Linard, C., Van Boeckel, T. P., Martin, V., Bhatt, S., Gething, P. W., Farrar, J. J., Hay, S. I., … Yu, H. (2014). Predicting the risk of avian influenza A H7N9 infection in live-poultry markets across Asia. *Nature communications*, *5*, 4116. <https://doi.org/10.1038/ncomms5116>
16. Li, X. L., Yang, Y., Sun, Y., Chen, W. J., Sun, R. X., Liu, K., Ma, M. J., Liang, S., Yao, H. W., Gray, G. C., Fang, L. Q., & Cao, W. C. (2015). Risk Distribution of Human Infections with Avian Influenza H7N9 and H5N1 virus in China. *Scientific reports*, *5*, 18610. <https://doi.org/10.1038/srep18610>
17. Chen, T., Guestrin, C., (2016)“XGBoost: A scalable tree boosting system,” in Proc. 22nd ACM SIGKDD Int. Conf. Knowl. Discovery Data Mining, pp. 785–794.<https://arxiv.org/abs/1603.02754>
18. Davagdorj, K., Pham, V. H., Theera-Umpon, N., & Ryu, K. H. (2020). XGBoost-Based Framework for Smoking-Induced Noncommunicable Disease Prediction. *International journal of environmental research and public health*, *17*(18), 6513. <https://doi.org/10.3390/ijerph17186513>
19. Guan, X., Zhang, B., Fu, M., Li, M., Yuan, X., Zhu, Y., Peng, J., Guo, H., & Lu, Y. (2021). Clinical and inflammatory features based machine learning model for fatal risk prediction of hospitalized COVID-19 patients: results from a retrospective cohort study. *Annals of medicine,* 53(1), 257–266. <https://doi.org/10.1080/07853890.2020.1868564>
20. Ogunleye, A., & Wang, Q. G. (2020). XGBoost Model for Chronic Kidney Disease Diagnosis. *IEEE/ACM transactions on computational biology and bioinformatics*, *17*(6), 2131–2140. https://doi.org/10.1109/TCBB.2019.2911071

**Table S1: Information of data sources in this study.**

| Variable | Source | Note | Website (Source) |
| --- | --- | --- | --- |
| Human MERS cases | World Health Organization, WHO | 1 981 cases with individual information | https://www.who.int/emergencies/mers-cov/en/ |
|  | Ministry of Health, Kingdom of Saudi Arabia | 457 cases added | https://www.moh.gov.sa/en/CoronaNew/PressReleases/Pages/default.aspx |
|  | Other news reports or articles | 12 cases added | Table S6 |
| MERS in animals | Food and Agriculture Organization of the United Nations, FAO | 39 locations have confirmed MERS-CoV cases in animals | <http://empres-i.fao.org/eipws3g/> |
|  | Literature | 66 papers confirmed positive test results in animals, 128 records added | Tables S7 |
| Population density | Socioeconomic Data and Applications Centre (SEDAC) of the International Geographic Science Information | A raster digital map with a resolution of 30 arc seconds | http://sedac.ciesin.columbia.edu/gpw/credits.jsp |
| Camel density | Office International Des Epizooties, OIE; Data on camel of Saudi Arabia |  | https://wahis.oie.int/#/dashboards/qd-dashboard;ttps://www.stats.gov.sa/en/22 |
|  | Food and Agriculture Organization of the United Nations, FAO | FAOSTAT country-level camel population data | http://www.fao.org/faostat/en |
| Meteorological data | National Oceanic and Atmospheric Administration, NOCC | Including average temperature and relative humidity, which 19 ecoclimatic variables (Bio1‒19) were created based on. | http://www.ncdc.noaa.gov/ |
| Elevation | The shuttle radar topography mission (SRTM) 90m DEM Digital Elevation Database |  | http://srtm.csi.cgiar.org |
| Land cover | European Aviation Administration | A raster digital map with a resolution of 300m | http://ionial.esrin.esa.int |
| Transportation | The OpenStreetMap project | Including railways and main roads | http://download.geofabrik.de/ |
| Locations of hospitals | The OpenStreetMap project |  | http://download.geofabrik.de/ |
| Map data | GADM database of Global Administrative Areas version 2.0 | Boundary data | http://www.gadm.org |

**Table S2: Description of 34 districts-level potential influencing factors used in the modelling efforts.**

| Variable | Description | Type | Website (Source) |
| --- | --- | --- | --- |
| BIO01 | Annual mean temperature (℃) | Continuous | http://www.ncdc.noaa.gov/ |
| BIO02 | Mean diurnal range (Mean of monthly (max temp-min temp)) (℃) | Continuous |
| BIO03 | Isothermality (BIO02/BIO07)(*100) | Continuous |
| BIO04 | Temperature seasonality (standard deviation*100) | Continuous |
| BIO05 | Max temperature of warmest month (℃) | Continuous |
| BIO06 | Min temperature of coldest month (℃) | Continuous |
| BIO07 | Annual range of temperature (BIO05-BIO06) (℃) | Continuous |
| BIO08 | Mean temperature of wettest quarter (℃) | Continuous |
| BIO09 | Mean temperature of driest quarter (℃) | Continuous |
| BIO10 | Mean temperature of warmest quarter (℃) | Continuous |
| BIO11 | Mean temperature of coldest quarter (℃) | Continuous |
| BIO12 | Annual precipitation (mm) | Continuous |
| BIO13 | Precipitation of wettest month (mm) | Continuous |
| BIO14 | Precipitation of driest month (mm) | Continuous |
| BIO15 | Precipitation seasonality(Coefficient of variation) | Continuous |
| BIO16 | Precipitation of wettest quarter (mm) | Continuous |
| BIO17 | Precipitation of driest quarter (mm) | Continuous |
| BIO18 | Precipitation of warmest quarter (mm) | Continuous |
| BIO19 | Precipitation of coldest quarter (mm) | Continuous |
| Cropland | Percentage coverage of cropland (%) | Continuous | http://ionial.esrin.esa.int |
| Forest | Percentage coverage of forest land (%) | Continuous |
| Grassland | Percentage coverage of grassland (%) | Continuous |
| Shrubland | Percentage coverage of shrubland (%) | Continuous |
| Wetland | Percentage coverage of wetland (%) | Continuous |
| Built-up land | Percentage coverage of built-up land (%) | Continuous |
| Bare land | Percentage coverage of bareland (%) | Continuous |
| Water body | Percentage coverage of water body (%) | Continuous |
| Ice land | Percentage coverage of ice land (%) | Continuous |
| Popular density | Density of human population (person per km^2^) | Continuous | http://sedac.ciesin.columbia.edu/gpw/credits.jsp |
| Camel density | Density of camel (head per km^2^) | Continuous | A combination data as described, |
| Elevation | Average altitude (m) | Continuous | http://srtm.csi.cgiar.org |
| Railway | The district is intersected by railway or not | Binary | http://download.geofabrik.de/ |
| Main road | The district is intersected by railway or not | Binary |
| Hospital | Number of hospitals in this district | Continuous |

**Table S3: Characteristics of confirmed MERS cases with different contact history from September 2012 to May 2020.**

|  | No. of cases (%) | | | *p*-value^§^ |
| --- | --- | --- | --- | --- |
|  | Total | With Animal Contact | Without Animal Contact |  |
| No. of confirmed cases | 2 450 | 356 | 1097 |  |
| Female | 751 (30.65) | 23 (6.46) | 461 (42.02) | <0.001^*^ |
| Age, years (median, IQR) | 53 (38–65) | 59 (47‒70) | 45 (32‒61) | <0.001^**^ |
| No. of deaths (CFR, %) | 802 (32.73) | 125 (35.11) | 267 (24.34) | <0.001^*^ |
| Healthcare worker | 335 (13.67) | 1 (0.28) | 284 (25.89) | <0.001^*^ |
| Asymptomatic infection | 201 (8.20) | 5 (1.40) | 177 (16.13) | <0.001^*^ |
| Presence of underlying condition | 1271 (51.88) | 274 (76.97) | 363 (33.09) | <0.001^*^ |
| Time from disease onset to diagnosis, days (median, IQR) | 5 (3–8) | 6.5 (4‒9) | 3 (2‒6) | <0.001^**^ |
| Time from disease onset to death, days (median, IQR) | 11 (7–17) | 11.5 (8-18) | 9 (5‒15) | <0.001^**^ |
| Year^†^ |  |  |  | 0.001^***^ |
| 2012-2013 | 178 (7.26) | 9 (9.1) | 90 (90.9) |  |
| 2014 | 652 (27.34) | 33 (11.0) | 267 (89.0) |  |
| 2015 | 680 (28.51) | 55 (11.2) | 437 (88.8) |  |
| 2016 | 254 (10.65) | 73 (47.4) | 81 (52.6) |  |
| 2017 | 250 (10.20) | 74 (44.8) | 91 (55.2) |  |
| 2018 | 150 (6.12) | 47 (58.0) | 34 (42.0) |  |
| 2019 | 226 (9.22) | 53 (38.3) | 87 (61.7) |  |
| 2020 | 60 (2.45) | 11 (52.4) | 10 (47.6) |  |

§ Statistical test between with or without animal contact. ^*^ Variables using χ² test, ^**^ Variables using Kruskal-Wallis test, ^***^ Cochran-Armitage trend test.

† Row percentages are shown for cases with or withnot animal contact.

Abbreviations: No, number; IQR, interquartile range; CFR, case fatality rate.

**Table S4: Relative importance (%) of predictors estimated for the persistence of MERS at county level by the boosted regression trees (BRT), random forests, support vector machines (SVM) and maximum entropy (MaxEnt).***

| Contributors | BRT | |  | Random Forests | |  | SVM | |  | MaxEnt | |
| --- | --- | --- | --- | --- | --- | --- | --- | --- | --- | --- | --- |
|  | Rank | Mean relative contribution (95% *CI*) |  | Rank | Mean gini coefficient (95% *CI*) |  | Rank | Mean relative contribution (95% *CI*) |  | Rank | Mean relative contribution (95% *CI*) |
| Percentage coverage of bareland | 1 | 30.06 (28.61–31.50) |  | 1 | 12.35 (10.43–14.26) |  | 1 | 11.31 (10.51–12.11) |  | 1 | 48.97 (46.55–51.39) |
| Percentage coverage of forest | 2 | 10.74 (9.56–11.91) |  | 4 | 9.32 (7.41–11.23) |  | 15 | 3.57 (3.22–3.92) |  | 3 | 4.22 (3.23–5.21) |
| Population density | 3 | 7.28 (6.69–7.87) |  | 6 | 7.38 (5.95–8.82) |  | 3 | 7.99 (7.11–8.86) |  | 16 | 0.57 (0.47–0.67) |
| Percentage coverage of cropland | 4 | 6.95 (6.07–7.84) |  | 2 | 10.28 (8.31–12.25) |  | 5 | 7.19 (6.71–7.67) |  | 7 | 2.03 (1.64–2.42) |
| Annual mean temperature (BIO 1) | 5 | 6.48 (6.06–6.90) |  | 8 | 6.91 (5.72–8.09) |  | 2 | 4.99 (4.43–5.55) |  | 5 | 3.67 (3.29–4.06) |
| Camel density | 6 | 6.20 (5.61–6.79) |  | 7 | 7.05 (5.01–9.09) |  | 13 | 4.94 (4.17–5.71) |  | 2 | 26.48 (23.29–29.68) |
| Max temperature of warmest month (BIO 5) | 7 | 4.63 (4.15–5.11) |  | 5 | 8.94 (6.97–10.92) |  | 10 | 4.94 (4.36–5.52) |  | 11 | 0.92 (0.67–1.17) |
| Annual precipitation (BIO 12) | 8 | 4.33 (3.91–4.75) |  | 3 | 9.41 (7.77–11.05) |  | 9 | 3.60 (3.26–3.93) |  | 9 | 1.23 (1.06–1.39) |
| Elevation | 9 | 4.17 (3.84–4.51) |  | 13 | 5.47 (4.34–6.59) |  | 7 | 4.78 (4.45–5.12) |  | 14 | 0.60 (0.51–0.70) |

*List the top nine factors which mean relative contributions greater than 4% in the BRT model.

**Table S5: Differential continuous variables included in models between positive (presence of MERS-CoV) and negative units (absence of MERS-CoV).**

| Variable | Absence (n=1820) | Presence (n=117) | P value* |
| --- | --- | --- | --- |
| Bareland (%, mean, 95%CI) | 15.80 (14.29–17.3) | 74.13 (67.56–80.69) | <0.001 |
| Forest (%, median, IQR) | 15.16 (1.92–38.95) | 0 (0–0.16) | <0.001 |
| Population density (log,100/km^2^, median, IQR) | 4.17 (4.09–4.25) | 3.58 (3.22–3.93) | 0.002 |
| Cropland (%, median, IQR) | 37.33 (10.68–62.01) | 0.48 (0.08–3.66) | <0.001 |
| Bio1 (℃, median, IQR) | 21.29 (15.2–26.31) | 26.48 (23.4–28.06) | <0.001 |
| Camel density (log, 1/km2, median, IQR) | -1.90 (-2.36–-1.53) | -1.68 (-2.13–-0.86) | <0.001 |
| Bio5 (℃, median, IQR) | 34.58 (34.35–34.81) | 40.70 (39.89–41.52) | <0.001 |
| Bio12 (mm, median, IQR) | 0.78 (0.33–1.29) | 0.13 (0.08–0.24) | <0.001 |
| Elevation (m, median, IQR) | 521.6 (217.82–1085.8) | 514.36 (154.56–811.12) | 0.033 |
| Bio3 (median, IQR) | 45.65 (34.49–61.6) | 41.01 (37.44–49.19) | 0.101 |
| Grassland (%, median, IQR) | 8.79 (1.44–23.2) | 1.13 (0.11–6.87) | <0.001 |
| Bio19 (mm, median, IQR) | 0.13 (0.02–0.29) | 0.03 (0.01–0.07) | <0.001 |
| Bio2 (℃, median, IQR) | 11.12 (9.37–12.72) | 13.11 (11.65–14.41) | <0.001 |
| Urban land (%, median, IQR) | 0.01 (0–0.33) | 0 (0–0.30) | 0.535 |
| Waterbody ((%, median, IQR) | 0.19 (0–1.69) | 0 (0–0) | <0.001 |
| Shrubland (%, median, IQR) | 15.80 (14.29–17.3) | 74.13 (67.56–80.69) | <0.001 |
| Wetland (%, median, IQR) | 15.16 (1.92–38.95) | 0 (0–0.16) | 0.011 |

* P values of Wilcoxon rank test between absence and presence units, except Bareland,Population density and Bio5 using T test.

**Table S6 The list of news reports and articles on human MERS cases records.**

| ID | Reference/Website(Source) |
| --- | --- |
| 1 | Reuss A, Litterst A, Drosten C, et al. Contact investigation for imported case of Middle East respiratory syndrome, Germany. Emerg Infect Dis. 2014;20(4):620-5. |
| 2 | Drosten C, Seilmaier M, Corman VM, et al. Clinical features and virological analysis of a case of Middle East respiratory syndrome coronavirus infection. Lancet Infect Dis. 2013;13(9):745-51. |
| 3 | Guery B, Poissy J, el Mansouf L, et al. Clinical features and viral diagnosis of two cases of infection with Middle East Respiratory Syndrome coronavirus: a report of nosocomial transmission. Lancet. 2013;381(9885):2265-72. |
| 4 | Hijawi B, Abdallat M, Sayaydeh A, et al. Novel coronavirus infections in Jordan, April 2012: epidemiological findings from a retrospective investigation. East Mediterr Health J. 2013;19 Suppl 1:S12-8. |
| 5 | Memish ZA, Zumla AI, Al-Hakeem RF, et al. Family cluster of Middle East respiratory syndrome coronavirus infections. N Engl J Med. 2013; 368(26):2487-94. |
| 6 | Zaki AM, van Boheemen S, Bestebroer TM, et al. Isolation of a novel coronavirus from a man with pneumonia in Saudi Arabia. N Engl J Med. 2012;367(19):1814-20. |
| 7 | https://www.cdc.gov/media/releases/2014/p0512-US-MERS.html(Accessed 8 February 2022) |
| 8 | http://www.flutrackers.com/forum/showthread.php?t=211669(Accessed 8 February 2022) |
| 9 | https://promedmail.org/promed-post/?id=2139610(Accessed 8 February 2022) |

**Table S7 The list of 66 papers for confirmed positive test of MERS in animals.**

| ID | Reference |
| --- | --- |
| 1 | Nowotny N , Kolodziejek J. Middle East respiratory syndrome coronavirus (MERS-CoV) in dromedary camels, Oman, 2013. Euro Surveill. 2014;19(16):20781. doi: 10.2807/1560-7917.es2014.19.16.20781. |
| 2 | Al Muhairi S, Al Hosani F , Eltahir YM, et al. Epidemiological investigation of Middle East respiratory syndrome coronavirus in dromedary camel farms linked with human infection in Abu Dhabi Emirate, United Arab Emirates. Virus Genes. 2016;52(6):848-854. doi: 10.1007/s11262-016-1367-1. |
| 3 | El-Kafrawy SA, Corman VM, Tolah AM et al.Enzootic patterns of Middle East respiratory syndrome coronavirus in imported African and local Arabian dromedary camels: a prospective genomic study. Lancet Planet Health. 2019;3(12):e521-e528. |
| 4 | Khalafalla AI, Lu XY, Al-Mubarak AI, Dalab AH, et al.MERS-CoV in Upper Respiratory Tract and Lungs of Dromedary Camels, Saudi Arabia, 2013-2014. Emerg Infect Dis. 2015;21(7):1153-8. |
| 5 | Kandeil A, Gomaa M, Nageh A, et al. Middle East Respiratory Syndrome Coronavirus (MERS-CoV) in Dromedary Camels in Africa and Middle East. Viruses. 2019;11(8):717. doi: 10.3390/v11080717. |
| 6 | Elfadil AA, Ahmed AG, Abdalla MO, et al. Epidemiological study of Middle East respiratory syndrome coronavirus infection in dromedary camels in Saudi Arabia, April-May 2015. Rev Sci Tech. 2018; 37(3):985-997. |
| 7 | Ommeh S, Zhang W, Zohaib Ali, et al.Genetic Evidence of Middle East Respiratory Syndrome Coronavirus (MERS-Cov) and Widespread Seroprevalence among Camels in Kenya. Virol Sin. 2018;33(6):484-492. |
| 8 | Kiambi S, Corman VM, Sitawa R, et al. Detection of distinct MERS-Coronavirus strains in dromedary camels from Kenya, 2017. Emerg Microbes Infect. 2018;7(1):195. |
| 9 | Zohaib A, Saqib M, Athar MA, et al. Countrywide Survey for MERS-Coronavirus Antibodies in Dromedaries and Humans in Pakistan. Virol Sin. 2018; 33(5):410-417. |
| 10 | Kasem S, Qasim I, Al-Doweriej A, Hashim O, et al.The prevalence of Middle East respiratory Syndrome coronavirus (MERS-CoV) infection in livestock and temporal relation to locations and seasons. J Infect Public Health. 2018;11(6):884-888. |
| 11 | Anthony SJ, Gilardi K, Menachery VD, et al.Further Evidence for Bats as the Evolutionary Source of Middle East Respiratory Syndrome Coronavirus. mBio. 2017; 8(2):e00373-17. doi: 10.1128/mBio.00373-17. |
| 12 | Miguel E, Perera RA, Baubekova A, et al. Absence of Middle East Respiratory Syndrome Coronavirus in Camelids, Kazakhstan, 2015. Emerg Infect Dis. 2016;22(3):555-7. |
| 13 | Gutiérrez C, Tejedor-Junco MT, González M, et al. Presence of antibodies but no evidence for circulation of MERS-CoV in dromedaries on the Canary Islands, 2015. Euro Surveill. 2015;20(37). |
| 14 | Shirato K, Azumano A, Nakao T, et al. Middle East respiratory syndrome coronavirus infection not found in camels in Japan. Jpn J Infect Dis. 2015;68(3):256-8. |
| 15 | Wernery U, Corman VM, Wong EY, et al. Acute middle East respiratory syndrome coronavirus infection in livestock Dromedaries, Dubai, 2014. Emerg Infect Dis. 2015;21(6):1019-22. |
| 16 | Woo PCY, Lau SKP, Teng JLL, et al. A novel astrovirus from dromedaries in the Middle East. J Gen Virol. 2015;96(9):2697-2707. |
| 17 | Woo PC, Lau SK, Li T, et al. A novel dromedary camel enterovirus in the family Picornaviridae from dromedaries in the Middle East. J Gen Virol. 2015; 96(7):1723-31. |
| 18 | Liu R, Wen Z, Wang J, et al. Absence of Middle East respiratory syndrome coronavirus in Bactrian camels in the West Inner Mongolia Autonomous Region of China: surveillance study results from July 2015. Emerg Microbes Infect. 2015;4(12):e73. |
| 19 | Wernery U, El Rasoul IH, Wong EY, et al. A phylogenetically distinct Middle East respiratory syndrome coronavirus detected in a dromedary calf from a closed dairy herd in Dubai with rising seroprevalence with age. Emerg Microbes Infect. 2015;4(12):e74. |
| 20 | Chan SM, Damdinjav B, Perera RA, et al. Absence of MERS-Coronavirus in Bactrian Camels, Southern Mongolia, November 2014. Emerg Infect Dis. 2015; 21(7):1269-71. |
| 21 | Paden CR, Yusof MFBM, Al Hammadi ZM,et al. Zoonotic origin and transmission of Middle East respiratory syndrome coronavirus in the UAE. Zoonoses Public Health. 2018;65(3):322-333. |
| 22 | Yusof MF, Queen K, Eltahir YM, et al. Diversity of Middle East respiratory syndrome coronaviruses in 109 dromedary camels based on full-genome sequencing, Abu Dhabi, United Arab Emirates. Emerg Microbes Infect. 2017;6(11):e101. |
| 23 | Li Y, Khalafalla AI, Paden CR, et al. Identification of diverse viruses in upper respiratory samples in dromedary camels from United Arab Emirates. PLoS One. 2017;12(9):e0184718. |
| 24 | Reusken CB, Schilp C, Raj VS, et al. MERS-CoV Infection of Alpaca in a Region Where MERS-CoV is Endemic. Emerg Infect Dis. 2016;22(6):1129-31. |
| 25 | Raj VS, Farag EA, Reusken CB, et al. Isolation of MERS coronavirus from a dromedary camel, Qatar, 2014. Emerg Infect Dis. 2014; 20(8):1339-42. |
| 26 | Reusken CB, Farag EA, Jonges M, et al. Middle East respiratory syndrome coronavirus (MERS-CoV) RNA and neutralising antibodies in milk collected according to local customs from dromedary camels, Qatar, April 2014. Euro Surveill. 2014;19(23):20829. |
| 27 | So RT, Perera RA, Oladipo JO, et al. Lack of serological evidence of Middle East respiratory syndrome coronavirus infection in virus exposed camel abattoir workers in Nigeria, 2016. Euro Surveill. 2018;23(32):1800175. |
| 28 | Falzarano D, Kamissoko B, de Wit E, et al. Dromedary camels in northern Mali have high seropositivity to MERS-CoV. One Health. 2017;3:41-43. |
| 29 | Kasem S, Qasim I, Al-Hufofi A, et al. Cross-sectional study of MERS-CoV-specific RNA and antibodies in animals that have had contact with MERS patients in Saudi Arabia. J Infect Public Health. 2018; 11(3):331-338. |
| 30 | Hemida MG, Alnaeem A, Chu DK, et al. Longitudinal study of Middle East Respiratory Syndrome coronavirus infection in dromedary camel herds in Saudi Arabia, 2014-2015. Emerg Microbes Infect. 2017; 6(6):e56. |
| 31 | Harrath R, Abu Duhier FM. Sero-prevalence of Middle East respiratory syndrome coronavirus (MERS-CoV) specific antibodies in dromedary camels in Tabuk, Saudi Arabia. J Med Virol. 2018;90(8):1285-1289. doi: 10.1002/jmv.25186. |
| 32 | Deem SL, Fèvre EM, Kinnaird M, et al. Serological Evidence of MERS-CoV Antibodies in Dromedary Camels (Camelus dromedaries) in Laikipia County, Kenya. PLoS One. 2015;10(10):e0140125. |
| 33 | van Doremalen N, Hijazeen ZS, Holloway P, et al. High Prevalence of Middle East Respiratory Coronavirus in Young Dromedary Camels in Jordan. Vector Borne Zoonotic Dis. 2017;17(2):155-159. |
| 34 | Harcourt JL, Rudoler N, Tamin A, et al. The prevalence of Middle East respiratory syndrome coronavirus (MERS-CoV) antibodies in dromedary camels in Israel. Zoonoses Public Health. 2018;65(6):749-754. |
| 35 | Woo PC, Lau SK, Wernery U, et al. Novel betacoronavirus in dromedaries of the Middle East, 2013. Emerg Infect Dis. 2014;20(4):560-72. |
| 36 | David D, Rotenberg D, Khinich E, et al. Middle East respiratory syndrome coronavirus specific antibodies in naturally exposed Israeli llamas, alpacas and camels. One Health. 2018;5:65-68. |
| 37 | Islam A, Epstein JH, Rostal MK, et al. Middle East Respiratory Syndrome Coronavirus Antibodies in Dromedary Camels, Bangladesh, 2015. Emerg Infect Dis. 2018;24(5):926-928. |
| 38 | Hemida MG, Perera RA, Al Jassim RA, et al. Seroepidemiology of Middle East respiratory syndrome (MERS) coronavirus in Saudi Arabia (1993) and Australia (2014) and characterisation of assay specificity. Euro Surveill. 2014;19(23):20828. |
| 39 | Munyua P, Corman VM, Bitek A, et al. No Serologic Evidence of Middle East Respiratory Syndrome Coronavirus Infection Among Camel Farmers Exposed to Highly Seropositive Camel Herds: A Household Linked Study, Kenya, 2013. Am J Trop Med Hyg. 2017; 96(6):1318-1324. |
| 40 | Memish ZA, Cotten M, Meyer B, et al. Human infection with MERS coronavirus after exposure to infected camels, Saudi Arabia, 2013. Emerg Infect Dis. 2014; 20(6):1012-5. |
| 41 | Al Hammadi ZM, Chu DK, Eltahir YM, et al. Asymptomatic MERS-CoV Infection in Humans Possibly Linked to Infected Dromedaries Imported from Oman to United Arab Emirates, May 2015. Emerg Infect Dis. 2015; 21(12):2197-200. |
| 42 | Chu DK, Poon LL, Gomaa MM, et al. MERS coronaviruses in dromedary camels, Egypt. Emerg Infect Dis. 2014;20(6):1049-53. |
| 43 | Hemida MG, Chu DK, Poon LL, et al. MERS coronavirus in dromedary camel herd, Saudi Arabia. Emerg Infect Dis. 2014; 20(7):1231-4. |
| 44 | Alexandersen S, Kobinger GP, Soule G, et al. Middle East respiratory syndrome coronavirus antibody reactors among camels in Dubai, United Arab Emirates, in 2005. Transbound Emerg Dis. 2014; 61(2):105-8. |
| 45 | Perera RA, Wang P, Gomaa MR, et al. Seroepidemiology for MERS coronavirus using microneutralisation and pseudoparticle virus neutralisation assays reveal a high prevalence of antibody in dromedary camels in Egypt, June 2013. Euro Surveill. 2013;18(36):pii=20574. |
| 46 | Memish ZA, Mishra N, Olival KJ, et al. Middle East respiratory syndrome coronavirus in bats, Saudi Arabia. Emerg Infect Dis. 2013;19(11):1819-23. |
| 47 | Hemida MG, Perera RA, Wang P, et al. Middle East Respiratory Syndrome (MERS) coronavirus seroprevalence in domestic livestock in Saudi Arabia, 2010 to 2013. Euro Surveill. 2013; 18(50):20659. |
| 48 | Reusken CB, Ababneh M, Raj VS, et al. Middle East Respiratory Syndrome coronavirus (MERS-CoV) serology in major livestock species in an affected region in Jordan, June to September 2013. Euro Surveill. 2013; 18(50):20662. |
| 49 | Saqib M, Sieberg A, Hussain MH, et al. Serologic Evidence for MERS-CoV Infection in Dromedary Camels, Punjab, Pakistan, 2012-2015. Emerg Infect Dis. 2017;23(3):550-551. |
| 50 | Müller MA, Corman VM, Jores J, et al. MERS coronavirus neutralizing antibodies in camels, Eastern Africa, 1983-1997. Emerg Infect Dis. 2014; 20(12):2093-5. |
| 51 | Reusken CB, Messadi L, Feyisa A, et al. Geographic distribution of MERS coronavirus among dromedary camels, Africa. Emerg Infect Dis. 2014; 20(8):1370-4. |
| 52 | Sabir JS, Lam TT, Ahmed MM, et al. Co-circulation of three camel coronavirus species and recombination of MERS-CoVs in Saudi Arabia. Science. 2016; 351(6268):81-4. |
| 53 | Ithete NL, Stoffberg S, Corman VM, et al. Close relative of human Middle East respiratory syndrome coronavirus in bat, South Africa. Emerg Infect Dis. 2013;19(10):1697-9. |
| 54 | Corman VM, Jores J, Meyer B, et al. Antibodies against MERS coronavirus in dromedary camels, Kenya, 1992-2013. Emerg Infect Dis. 2014;20(8):1319-22. |
| 55 | Ferguson NM, Van Kerkhove MD. Identification of MERS-CoV in dromedary camels. Lancet Infect Dis. 2014; 14(2):93-4. |
| 56 | Meyer B, Müller MA, Corman VM, et al. Antibodies against MERS coronavirus in dromedary camels, United Arab Emirates, 2003 and 2013. Emerg Infect Dis. 2014; 20(4):552-9. |
| 57 | Yusof MF, Eltahir YM, Serhan WS, et al. Prevalence of Middle East respiratory syndrome coronavirus (MERS-CoV) in dromedary camels in Abu Dhabi Emirate, United Arab Emirates. Virus Genes. 2015; 50(3):509-13. |
| 58 | Alagaili AN, Briese T, Mishra N, et al. Middle East respiratory syndrome coronavirus infection in dromedary camels in Saudi Arabia. mBio. 2014;5(2):e00884-14. |
| 59 | Farag EA, Reusken CB, Haagmans BL, et al. High proportion of MERS-CoV shedding dromedaries at slaughterhouse with a potential epidemiological link to human cases, Qatar 2014. Infect Ecol Epidemiol. 2015;5:28305. |
| 60 | Gossner C, Danielson N, Gervelmeyer A, et al. Human-Dromedary Camel Interactions and the Risk of Acquiring Zoonotic Middle East Respiratory Syndrome Coronavirus Infection. Zoonoses Public Health. 2016; 63(1):1-9. |
| 61 | Miguel E, Chevalier V, Ayelet G, et al. Risk factors for MERS coronavirus infection in dromedary camels in Burkina Faso, Ethiopia, and Morocco, 2015. Euro Surveill. 2017;22(13):30498. |
| 62 | Ali MA, Shehata MM, Gomaa MR, et al. Systematic, active surveillance for Middle East respiratory syndrome coronavirus in camels in Egypt. Emerg Microbes Infect. 2017; 6(1):e1. |
| 63 | Lau SK, Wernery R, Wong EY, et al. Polyphyletic origin of MERS coronaviruses and isolation of a novel clade A strain from dromedary camels in the United Arab Emirates. Emerg Microbes Infect. 2016;5(12):e128. |
| 64 | Ali M, El-Shesheny R, Kandeil A, et al. Cross-sectional surveillance of Middle East respiratory syndrome coronavirus (MERS-CoV) in dromedary camels and other mammals in Egypt, August 2015 to January 2016. Euro Surveill. 2017; 22(11):30487. |
| 65 | Chu DK, Oladipo JO, Perera RA, et al. Middle East respiratory syndrome coronavirus (MERS-CoV) in dromedary camels in Nigeria, 2015. Euro Surveill. 2015; 20(49). |
| 66 | Haagmans BL, Al Dhahiry SH, Reusken CB, et al. Middle East respiratory syndrome coronavirus in dromedary camels: an outbreak investigation. Lancet Infect Dis. 2014;14(2):140-5. |

**Figure S1: Distribution of countries with reported human MERS cases in the world during 2012**–**2020.** Countries were colored according to the dominant transmission type: locally animal-related and transmitted, locally animal-related and non-transmitted, imported and transmitted, imported and non-transmitted.
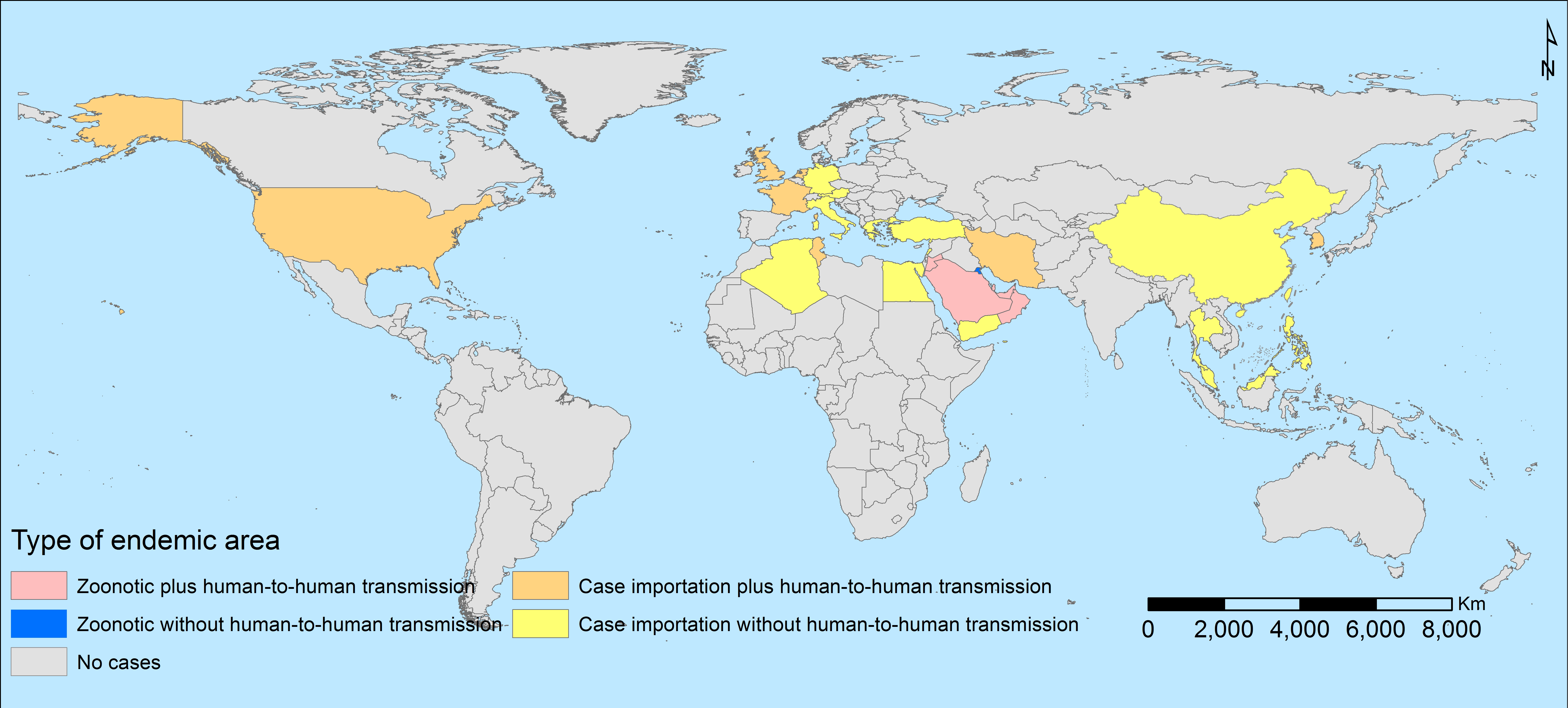


**Figure S2: Scatter plot and R-square value of 4 different imputation method.** (A) Classification and regression trees, (B) Random forest imputations, (C) Predictive mean matching and (D) Linear regression.


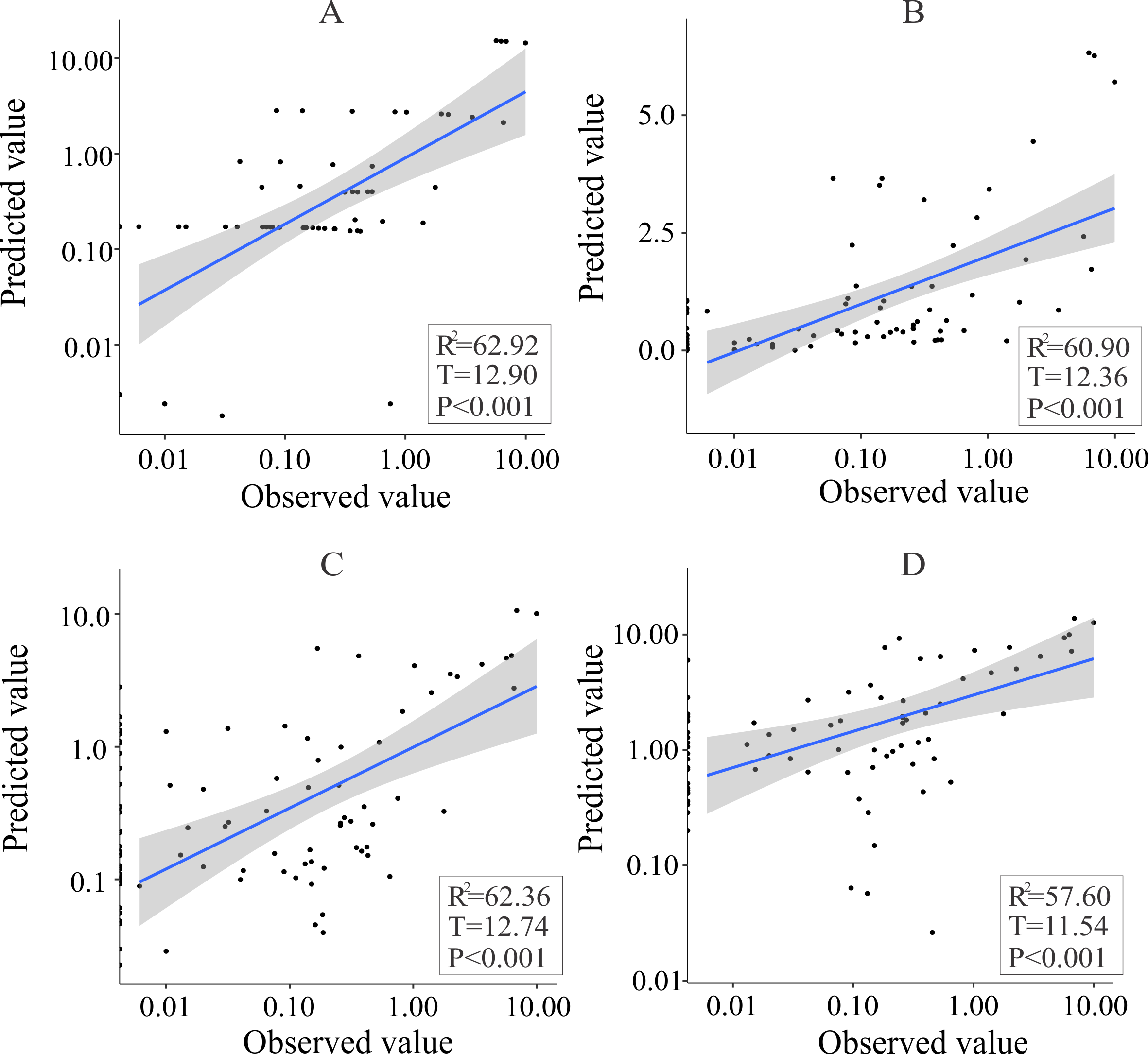


**Figure S3: Spatial distribution of camel density for the original data (A) and the average of 100 imputed data sets generated from a CART model (B).** In panel A, areas filled with gray have no data, and areas shaded with stripes have data at the country level.

**
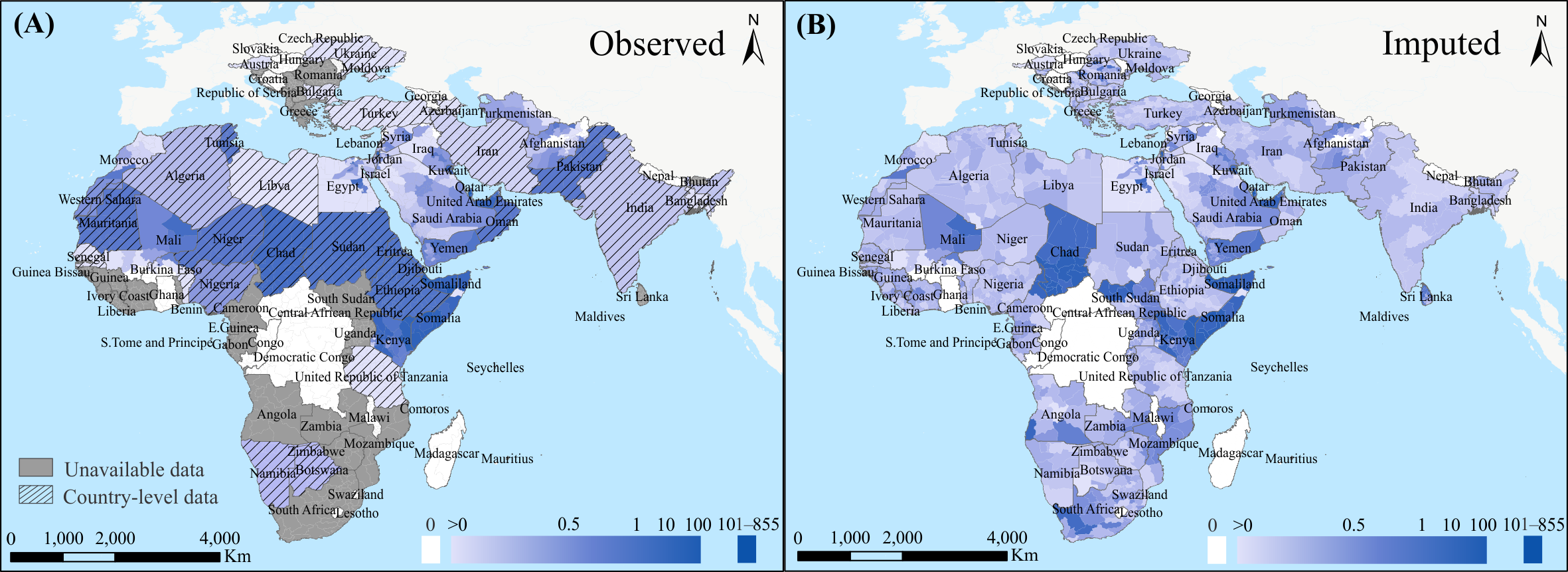
**

**Figure S4: Correlation Matrix for 34 variables.** Heatmap representing the correlation between features using Spearman correlation coefficient. Red and blue colors in the plot represent the positive and negative monotonic relationship respectively. The absolute value of the correlation coefficient was represented by the size of the circle, and the exact value shown in the lower triangle.


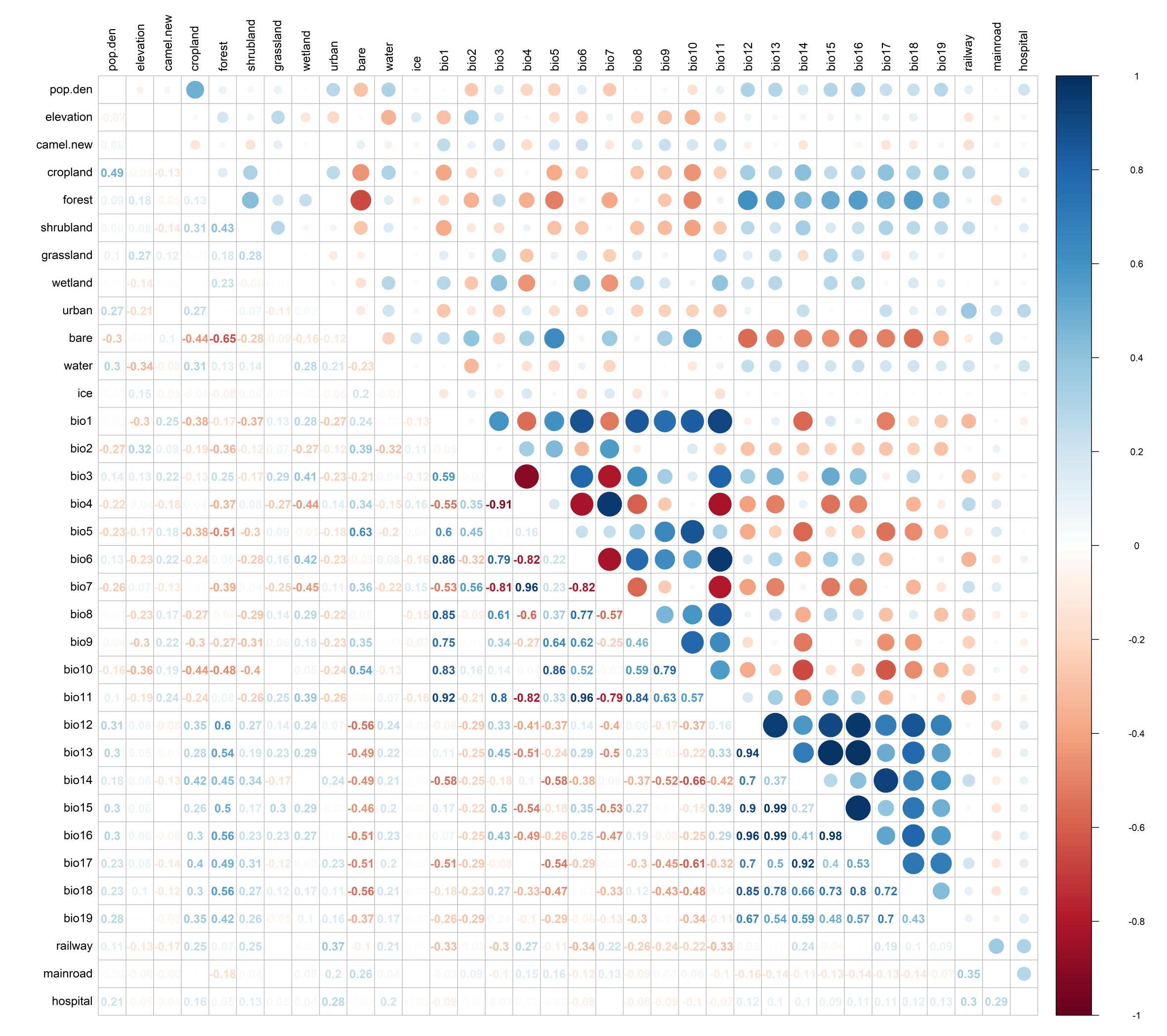


**Figure S5: ROC curves and AUC values of training and testing data sets in different machine learning models.** Random Forest (RF), Support Vector Machines (SVM), Boosted Regression Trees (BRT) and stacking model were included.


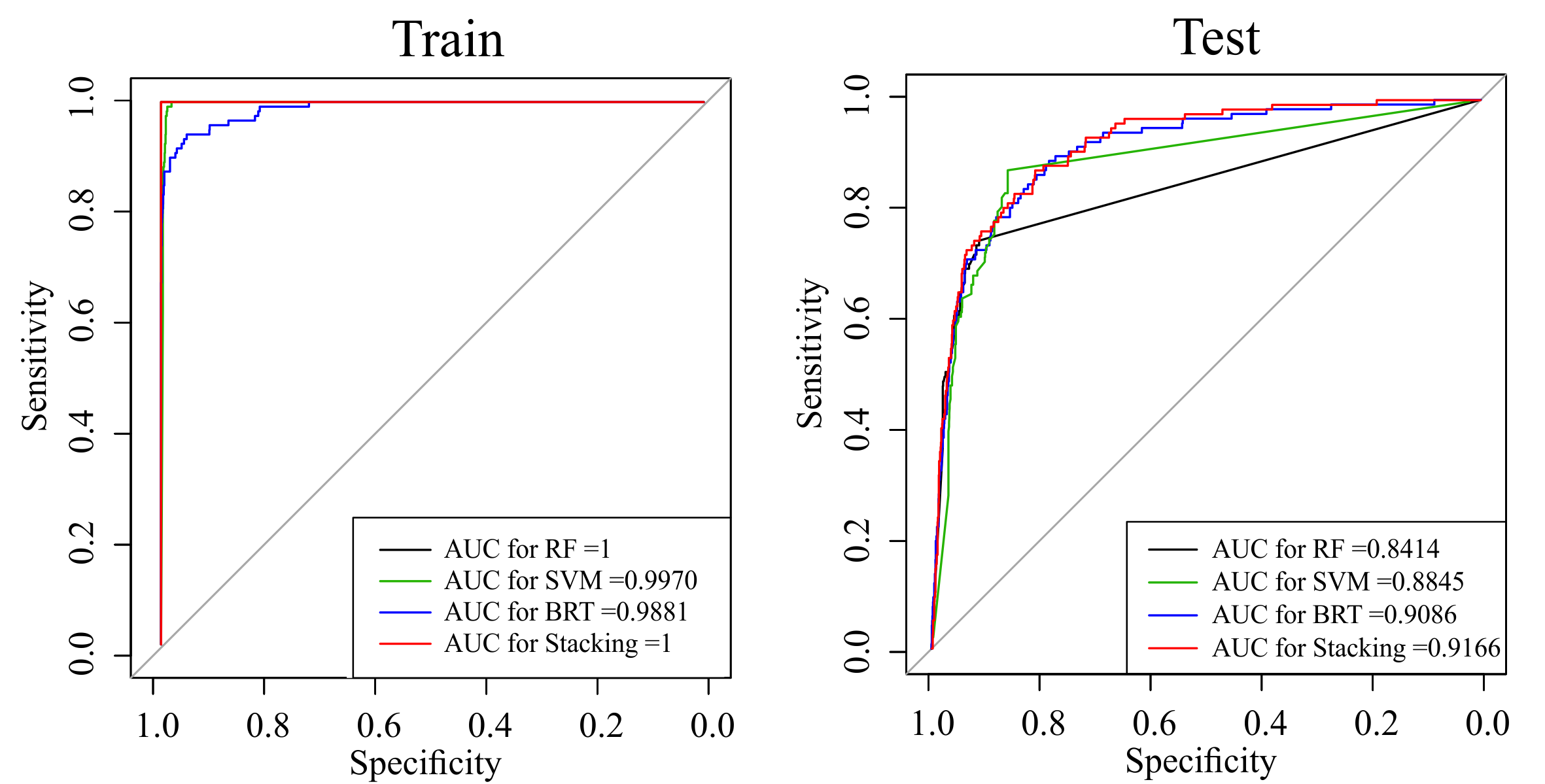


***** Average ROC curve and mean AUC value of train data and test data are shown after combined 100 individual models.

Abbreviations: ROC, receiver operating characteristic; AUC, area under the ROC curve.

**Figure S6: Performance of 4 models on training data in respect to different probability cutoff value.** (A) stacking model; (B) Boosted Regression Trees (BRT); (C) Support Vector Machines (SVM); (D) Random Forest (RF).

Colorful lines indicate the corresponding value of the cutoff p value. Dot colored with red indicates the highest F1 score with chosen cutoff p value.


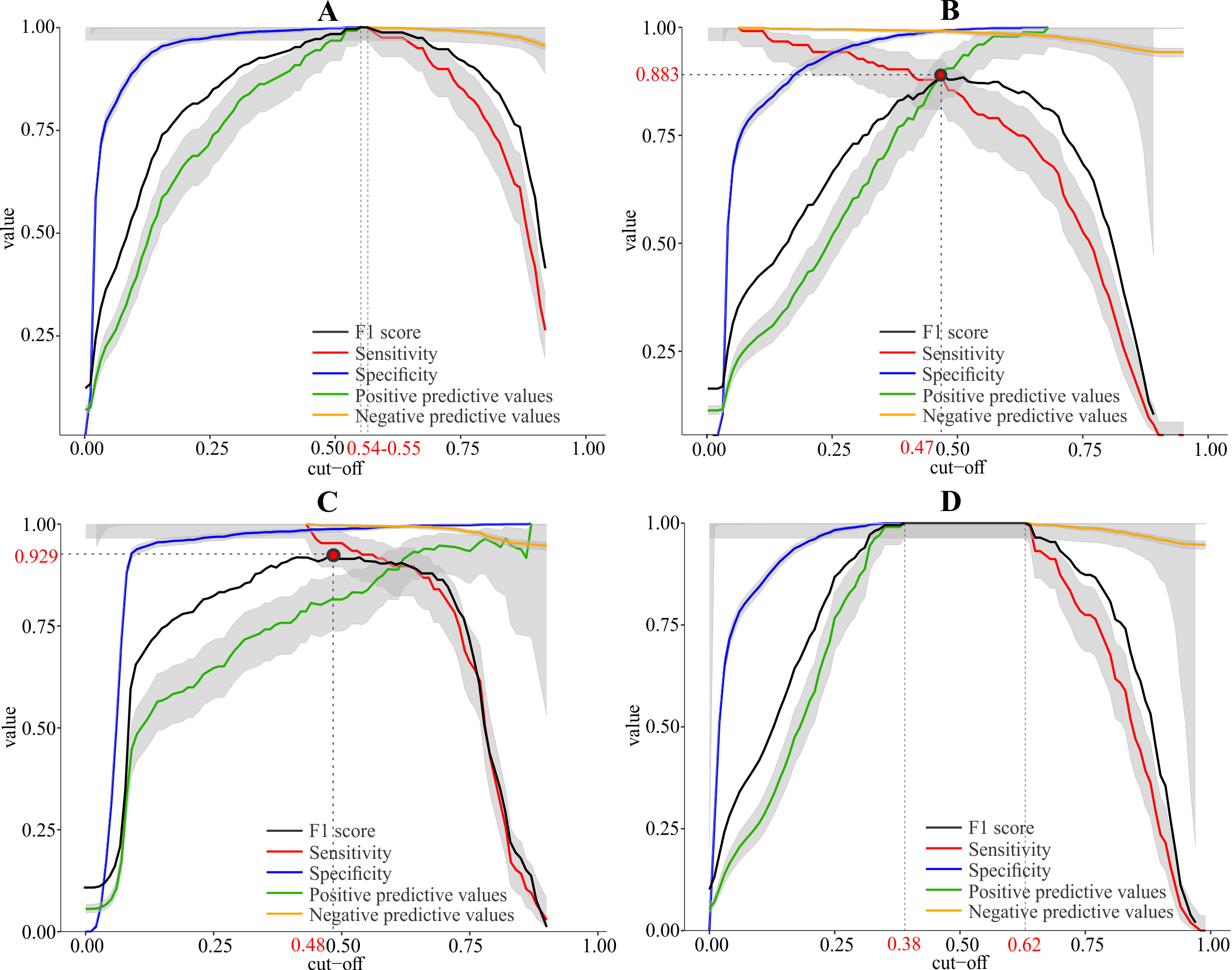


**Figure S7: Risk assessment and relative variable importance by MaxEnt model**. (A) ROC curves and AUC values of training and testing data sets in MaxEnt model. (B) The efficiency estimation of variables in MaxEnt model with all 20 factors included for analysis. Points represent the mean value, lines across on them represent the 95% confidence interval.(C) mapped model-predicted risks of human infections with MERS-CoV on data in our study.


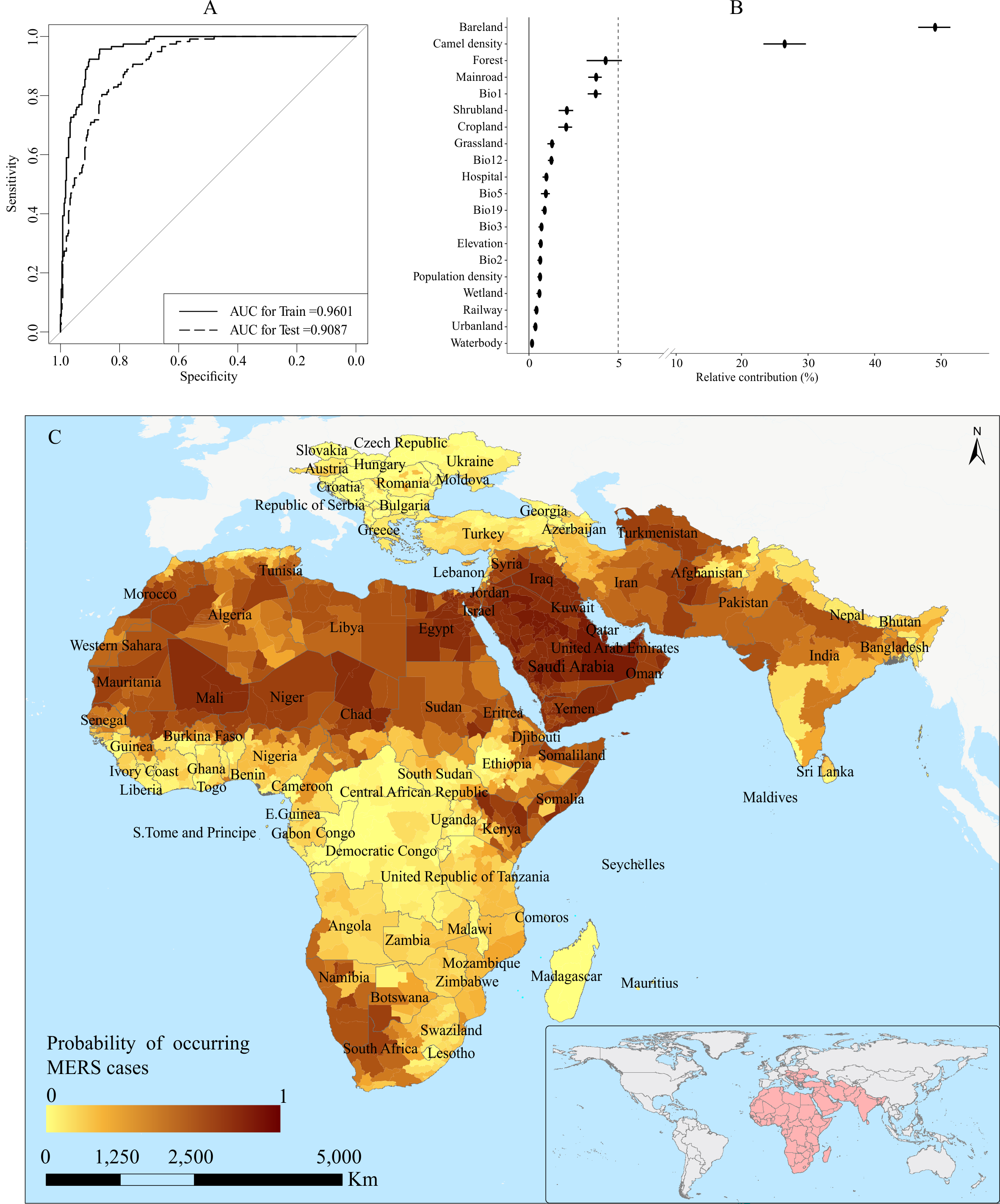


**Figure S8: Cutoff value of importance variables with relative contribution over 5% in BRT model based on Yuden index.** (A) Percent coverage of bareland: (B) Percent coverage of forest; (C) Population density; (D) Percent coverage of cropland; (E) Bio1: Annual mean temperature; (F) Camel density. Dot colored with red indicates the highest Yuden index with chosen cutoff value.


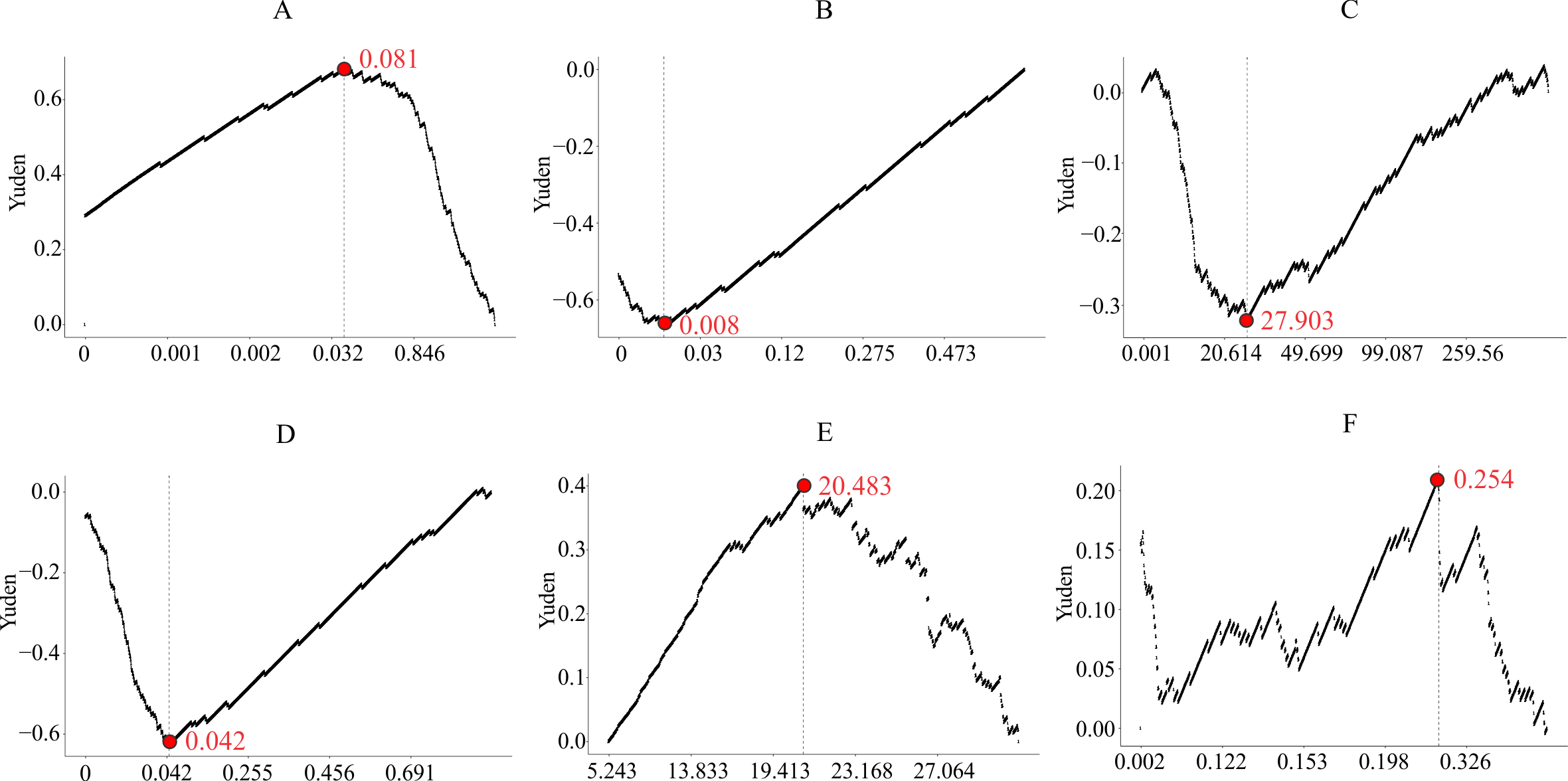

Supplement: Supplementary file 1 — Supporting Information [file TBED-69-e2122-s001.doc]
